# Estimating global numbers of farmed fishes killed for food annually from 1990 to 2019

**DOI:** 10.1017/awf.2023.4

**Published:** 2023-02-06

**Authors:** Alison Mood, Elena Lara, Natasha K Boyland, Phil Brooke

**Affiliations:** 1 Fishcount.org.uk; 2 Compassion in World Farming International, River Court, Mill Lane, Godalming GU7 1EZ, UK

**Keywords:** animal welfare, aquaculture, estimated numbers, farmed fish, fish slaughter, fish welfare

## Abstract

Global farmed finfish production increased from 9 to 56 million tonnes between 1990 and 2019. Although finfishes are now widely recognised as sentient beings, production is still being quantified as biomass rather than number of individuals (in contrast to farmed mammals and birds). Here, we estimate the global number of farmed finfishes slaughtered using FAO aquaculture production tonnages (1990–2019 data) and estimates of individual weight at killing (determined from internet searches at species and country level where possible). We relate these numbers to knowledge on humane slaughter, animal welfare law, and certification schemes. Since 1990, farmed finfish numbers killed annually for food have increased nine-fold, to 124 billion (1.24 × 10^11^, range 78–171 billion) in 2019. This figure does not represent the total number farmed (due to mortalities during rearing and non-food production) and is expected to increase as aquaculture expands. Our estimates indicate that farmed finfishes now outnumber the 80 billion farmed birds and mammals killed globally each year for food. The majority are produced in Asia. Inhumane slaughter practices cause suffering for most farmed finfishes. Most, 70–72%, have no legal welfare protection, and less than 1% have any fish-specific legal protection, at slaughter. The main global certification schemes in 2013–2015 accounted for 2% of slaughtered farmed finfishes. Fishes for which species-specific parameters for automated humane stunning are published comprise 20–24%. As the dominant taxa of farmed vertebrates, finfishes would benefit from better welfare if species-specific humane slaughter was defined and incorporated into laws and certification schemes.

## Introduction

The magnitude of an animal welfare problem may be measured as the product of the severity, duration and numbers of animals affected (World Society for the Protection of Animals [WSPA] 2003). This study estimates the number of farmed fishes killed for food in global aquaculture from 1990 to 2019, towards an assessment of the scale of the fish welfare issue in this rapidly growing industry.

Aquaculture is important for the food, nutrition and employment of millions of people, according to the Food and Agriculture Organisation of the United Nations (FAO 2018). The FAO publishes statistics for global farmed finfish production, which reached 56 million tonnes in 2019 (FAO 2021). Despite wide acceptance of fish sentience, these statistics are given only in tonnages and not numbers, in contrast with FAO statistics for farmed birds and mammals which are given in both (FAO 2020a). Production tonnages are unlikely to be a good indication of farmed fish numbers since fish size, at slaughter, varies greatly between species.

Research evidence confirms that fish species are capable of nociception (detection of painful stimuli) and appear to experience a negative affective state following noxious stimulation (Sneddon 2009). Acceptance of fish sentience is implicit in the farmed fish welfare codes of the World Organisation for Animal Health, founded as OIE (2022a), and in much national legislation, as will be shown.

Fish farming can compromise the health and welfare of fishes due to, for example, disease and lice infestation; social stress and aggression; handling and transport; feed withdrawal; inability to express natural behaviours; and inhumane methods of slaughter (Ashley 2007). Unfortunately, scientific information on the welfare needs of most fish species reared for food is limited, including their physical requirements, ethological needs and parameters for humane slaughter (Humane Slaughter Association [HSA] 2018; Franks *et al.* 2021).

The OIE’s guidelines to protect the welfare of farmed fishes during slaughter (2022b), last adopted in 2012, state that fishes should be stunned before killing, to ensure immediate loss of consciousness, and killed before consciousness is recovered if the stunning is not irreversible. Recommended stunning methods, if applied correctly, are percussive stunning; spiking (ike jime); shooting; and electrical stunning (in-water or semi-dry). Stunning can be applied manually, e.g. a manual percussive stun with a club, or with specially developed percussive or electrical stunning equipment (OIE 2022b).

The following killing methods are not recommended by OIE because they result in poor welfare: asphyxiation by removal from water; chilling with ice in water; killing with CO_2_; exsanguination without stunning; and salt or ammonia baths.

Regardless of the available guidance from OIE on fish slaughter, an overwhelming majority of fishes farmed throughout the world are currently killed with little or no consideration for their welfare, with most killed by asphyxia in air or ice slurry, without prior stunning (Lines & Spence 2014).

These animals suffer very poor welfare in large numbers. Mood and Brooke (2012) estimated that 37–122 billion farmed fishes were killed for food in 2010 (not peer-reviewed). This was based on estimated mean weights (EMWs), derived from internet-sourced slaughter weights, combined with FAO tonnages. The data, including EMWs, were published in the public domain (fishcount.org.uk).

More recently, Franks *et al.* (2021) obtained a range of 59–129 billion farmed aquatic vertebrates for 2018, mostly fishes, using estimates of mean weights extrapolated from maximum weights obtained from fishbase.org. Their model incorporated 48 fishcount EMWs in calibrating relationships between maximum weights and slaughter weights.

The current study aims to provide a new, refined and extended estimate, obtaining a more complete set of EMWs based on reported slaughter weights and incorporating new data and geographical variation. These updated EMWs are used to show changes in numbers over time, with estimated fish numbers for:

- Species farmed in the highest numbers, globally, regionally and in the main countries;
- Species for which stunning parameters for automated humane slaughter are published, based on Spence (2014; cited in HSA 2018);
- Fishes farmed in countries with some stated legal protection at slaughter; and
- Fishes farmed within aquaculture certification schemes, based on Potts *et al.* (2016).

## Materials and methods

This study used EMWs for farmed fish species at slaughter to estimate numbers killed for food globally from FAO aquaculture production tonnages, annually, over the period from 1990 to 2019. All data were stored on a MySQL database, with calculations performed in MySQL code and Microsoft Excel.

### FAO production tonnage data

A list of farmed finfish species categories, and their aquaculture production tonnage for each country in each year between 1990 and 2019, was obtained from the FAO (2021) using ‘FishStatJ’ software. The time-series filter feature was used to select all data for the species main group ‘PISCES.’

The FAO is the only source of global aquaculture production statistics, which represent a unique global asset for sector analysis and monitoring (FAO 2018). These are based on national statistics supplied by countries, usually based on sample-surveys (FAO 2020b). A limitation is that not all species are reported separately; with some reported by genus, family or vague species groupings such as ‘Freshwater fishes nei’. ‘Nei’ is short for ‘not elsewhere included,’ a term used by FAO in the absence of species information. The FAO collaborates with countries to improve the level of species breakdown and quality of their statistics (FAO 2018). Some potential sources of inaccuracy therein are discussed later.

As a separate process from estimating fish numbers, as described in the sections below, production tonnage in 2019 was compared to that in 1990.

### Collection of data for EMWs

EMWs were based on farmed fish slaughter sizes (e.g. ‘harvest’ or market sizes), obtained from internet searches in Google and Google Scholar. In addition, fish size data from Mood and Brooke (2012), obtained in a similar way, were also included.

Search terms included the word ‘farmed’ or ‘cultured’, followed by the scientific name (in quotes), and one of the following (in quotes): ‘harvest size’, ‘market size’, ‘average size’ ‘mean size’, ‘marketable size’, ‘commercial size’ or ‘table size’ and a similar list with ‘weight’ substituted for ‘size’. For example, “farmed ‘*Salmo salar*’ ‘harvest weight.’” Some searches additionally included the name of the highest producing country in 2019. If the above did not return sufficient suitable data, then additional search terms were used, these being modifications of the initial search terms, e.g. with ‘kg’ substituted for ‘size’ or ‘weight.’

Only sizes relating to farmed fishes at capture or slaughter were collected. Some references, e.g. census publications, reported weights for multiple species. One fish weight was obtained from another researcher.

The collected fish sizes were reviewed for any that were evidently untypical, in order to eliminate uncommon sizes from these estimates. Where systematic census data were available (see following section), any corresponding data from other sources were also eliminated.

### EMWs for single-species categories

Since global mean slaughter weights are not included in FAO fishery statistics, EMWs were extrapolated from other data. Not all types of data were considered equally likely to produce a reliable EMW. The collected data were each categorised as one of the following six types of fish weight, some advantages and disadvantages of which are as follows:

- Average or mean weights. Mean weights are the most relevant data and include those from census data, considered the most reliable data. Weights reported simply as ‘average’ weights are assumed to represent mean weights.
- Simple weight ranges. These are likely to span the mean weight, but may be imprecise, i.e. give a wide range.
- Usual or most common weights. These terms describe the mode rather the mean, which can be smaller or larger. For example, small numbers of fishes above the usual size will tend to increase the mean weight above the modal weight, in which case fish numbers based on the lower mode would be over-estimated. Most farmed barramundi (*Lates calcarifer*) sold in Asia weigh 500–900 g, although small numbers of larger fish (1–3 kg) are also sold (FAO 2022a).
- Preferred or optimal weights. These could represent the most usual sizes but could also be biased if referring to larger sizes that attract a higher price.
- Common or typical weights. These may not be the usual or most common ones.
- Weights from a marketing website or report. Similarly, these may not be the most common weights. These are also assumed to represent weights of a whole fish, rather than dressed weights unless otherwise stated, though it is not certain this is always the case.

Fish size data for species were considered more reliable when they related to the country being estimated, and most reliable when obtained from an aquaculture census, i.e. a systematic government survey of a country’s aquaculture.

Since the collected data were not considered equally reliable, the derivation of EMWs entailed selection of data, achieved by means of a data ranking system.

EMWs were obtained for one species and country at a time. The fish size data to include in each EMW were selected via the data ranking system. There were five different data ranking systems (Table 1), one for the main estimate and one for each of four alternative estimates (A1 to A4) in the sensitivity analysis. For each EMW, only data of the highest ranking available were used. Lower and upper EMWs were calculated from the outside range of selected fish sizes.

**Table 1.** Ranking of fish size data used to estimate mean weight of a species in a country

Geographic region of data	Type of fish weight	Ranking of data in estimate				
		Main	A1	A2	A3	A4
Country being estimated	Average, mean, usual, modal, preferred or optimal weights or simple weight ranges	1	NA	NA	NA	NA
Continent of above	As above	1	NA	NA	NA	NA
Global and primary country	As above	2	1	1	1	1
Non-primary country	As above	3	1	1	1	2
Any	Common/typical	4	1	1	2	3
Any	Marketing website/report	5	1	2	3	4

Ranking of fish size data in the main estimate and alternative estimates A1 to A4. ‘1’ indicates the highest ranked data. Higher ranked data is used in preference to lower ranked data, where available. Fish sizes are ranked according to type of fish weight and country to which they refer. In the main estimate, highest ranked data includes average weights for the country being estimated. Second highest ranked data includes average weights for the primary producing country. For example, the highest ranked data for Atlantic salmon in the UK includes average weights for the UK, and the second highest ranked data includes average weights for Norway, the largest salmon producer by tonnage.

Fish size data are ranked according to the type of fish size and country to which they apply. The main estimate ranks the first four types of data (average, usual, preferred or optimal weights or simple weight ranges) equally since, in the absence of census data, it is not possible to determine which are the most reliable and representative and including more data should increase the sub-sectors represented. Common weights are ranked lower, and weights from a marketing website/report lower still.

In the main estimate, for any species and country, the ranking system works as follows (Table 1):

- Data from that country (including average, usual, preferred or optimal weights or simple weight ranges);
- Similar data from the top producing country or those categorised as global sizes;
- Similar data from countries other than the above;
- Common weights from any country;
- Marketing website/report weights from any country.

Census data were not identified separately in the data ranking systems (Table 1). Instead, they were effectively ranked highest, in all estimates, by the elimination of corresponding data from other sources during collection of fish size data.

EMWs were usually derived as a range, since fish weights are more often reported as such and many EMWs incorporate more than one report. Global EMWs were later back-calculated from total estimated numbers for a species (see following section) and its global production tonnage.

### Estimates of fish numbers for single-species EMWs

For each species with fish size data, fish numbers were calculated by dividing the production tonnage for each country by the respective upper and lower EMW, and then totalled to obtain a global number range for the species. In turn, these were then used to calculate global EMWs (see above).

### Estimates for multi-species categories

EMWs for multi-species categories, each relating to a genus, were obtained by combining the outer ranges of two global single-species EMWs, these being for the smallest and largest farmed species in that category for which fish size data were obtained. The smallest and largest species were determined as those with respectively the smallest lower EMW and the greatest upper EMW.

Where data were available for the group generally, then multi-species EMWs were based on this using the same method as for single-species EMWs. Numbers were calculated from the multi-species EMW and the global tonnage.

### Estimates for species categories without an EMW

For species categories for which no EMW was obtained, a generic estimated mean weight (GEMW) was used instead. Species were divided into groups, usually by taxonomic order. GEMWs were extrapolated from the overall average individual fish weight range for all single-species categories with an EMW, within each species group.

### Estimate totals

The total global estimate for each year was obtained as the sum of all estimates for single- and multi-species categories, together with those based on GEMWs. In addition to the total global estimate, and the global estimates for species, estimates were calculated for each country and for each year between 1990 and 2019.

### Sensitivity analysis

Four alternative estimates, A1 to A4, were also performed for 2019 to test the sensitivity of the estimate to changes in the data ranking system (Table 1).

Two further estimates, A5 and A6, concern fish market weights. In the main estimate, it was assumed that these represent a whole fish unless stated otherwise. A5 and A6 tested the alternative assumption that such weights were in fact headed and gutted, converting them to liveweights (the method being otherwise identical to that for the main estimate).

Reported gutted and headless sizes include 52 and 53% of live weight for farmed silver barb (*Barbonymus gonionotus* or *Puntius gonionotus*) and Nile tilapia (*Oreochromis niloticus*), respectively (Sahu *et al.* 2017), and 64% for farmed medium carp (*Hypselobarbus pulchellus*) (Raghunath *et al.* 2016). Based on these, A5 and A6 assumed that market weights represent 64 and 52% of the fish liveweight, respectively. A5 and A6 used the same data ranking system as for the main estimate.

### Analysis of fish weights obtained from a census

Mean fish slaughter weights obtained from aquaculture censuses were analysed for change over time. They were also compared with any corresponding data obtained from other sources, as a test of reliability.

### Slaughter analysis

The parameters required to stun fishes effectively, using stunning machines, have been published for some species (Spence 2014; cited in HSA 2018). The total estimated number of farmed fishes that would benefit from the application of these parameters was calculated.

An estimate of farmed fish numbers with, and without, some legal protection during slaughter, according to the wording of law, was made for 28 non-EU countries and the EU27 countries combined. Firstly, reference was made to the Animal Protection Index report for each country analysed, published by World Animal Protection (2020), which give the country’s main animal protection laws. Internet searches were then performed, in Google, to locate relevant laws. These search terms were used: ‘animal welfare law’ or ‘animal law’ followed by the name of the country; or name of the law. Laws were translated into English, as necessary, using Google Translate. The legal texts obtained were then studied for information on the species covered and any protection applicable to farmed fishes, especially during slaughter.

Fish numbers reared on farms within certification schemes in 2013–2015 were estimated, using certified production tonnages, for 2013, 2014 or 2015, for each of the main global certification schemes from Potts *et al.* (2016). Global EMWs (see *EMWs for single-species categories* above) were used to convert these tonnages to numbers. These schemes have developed, or are developing, welfare standards at slaughter (discussed later).

## Results

### FAO production tonnage data

Between 1990 and 2019, annual global farmed finfish production rose 6.5-fold from 9 to 56 million tonnes (Table 2). Asia accounted for 88% of this increase; China alone for 47%. Africa showed the greatest relative growth, increasing 29-fold.

**Table 2.** Global farmed finfish production by continent in 1990 and 2019 (FAO 2021)

Continent	1990 Production (‘000 t)	2019 Production (‘000 t)	Fold increase	1990 Percent share %	2019 Percent share %
Asia	7,319	49,082	6.7	84	87
Europe	954	2,588	2.7	11	5
Americas	319	2,303	7.2	4	4
Africa	78	2,265	28.9	1	4
Oceania	7	89	13.6	0.1	0.2
Total	8,676	56,327	6.5	100	100

Comparing global production in 1990 and 2019, the great majority of the increase, 71%, was due to growth in existing sectors, e.g. grass carp (*Ctenopharyngodon idellus*) farming in China and catla (*Catla catla*) farming in India. A further 22% was due to the expansion of species into additional countries, where both species and country were previously reported in finfish aquaculture in 1990, e.g. striped catfish (*Pangasianodon hypophthalmus* or *Pangasius hypophthalmus*). Seven percent was due to the farming of species not previously reported, e.g. yellow catfish (*Pelteobagrus fulvidraco*), and less than 0.2% was due to farming in countries which had not previously reported any finfish aquaculture.

### Collection of data for EMWs

A total of 237 fish sizes were obtained from 159 reference documents. Eight were discarded at the beginning, including three small sizes for sewage-fed aquaculture in West Bengal, that reportedly produces relatively small tonnages of 25,000 per year (Singh 2019), and five sizes for which corresponding census data were available.

After further exclusions by the ranking system, the main estimate used 196 fish weights, sources of which are summarised in Table S1 of the supplementary material. These data, which included 84 fish weights from Mood and Brooke (2012), were categorised as follows: 46 mean or average weights, 78 simple weight ranges, 57 usual or most common weights; nine preferred or optimal weights; three common or typical weights and three weights from a marketing website/report. Of these, six fish sizes related to a genus or family, rather than a single species.

### EMWs for single-species categories

Single-species EMWs were obtained for 91 species, comprising 81% of total finfish tonnage for 2019. Cases where EMWs for a country differed from the global EMW (following section) are shown in Table S2 of the supplementary material. Single-species EMWs based on rank 1 and rank 2 data (Table 1) accounted for, respectively, 50 and 25% of the total estimate by tonnage (Table S3 of the supplementary material).

### Estimates of fish numbers for single-species EMWs

A total estimated range of 63–126 billion fishes was obtained for the 91 single-species categories with an EMW. Estimated numbers and global EMWs for these are shown in Table S4 of the supplementary material. Estimates for the top 38 species categories are shown in Table 3, of which 31 are single-species categories with an EMW.

**Table 3.** Estimated global number ranges for the top 38 fish species killed for food (2019) by estimate mid-point

Rank	FAO species category (s*cientific name*) MS = multi-species category	Stun parameters?	Production in ‘000 t	% Total fish t	Estimated global mean weight range (g)		Estimated number range (millions) to 2 sig fig		
					Lower	Upper	Lower	Upper	Midp’nt
1	Pond loach (*Misgurnus anguillicaudatus*)		358	1	10	26	14,000	36,000	25,000
2	Carassius spp (*Carassius* spp) MS		2,756	5	150	500	5,500	18,000	12,000
3	Nile tilapia (*Oreochromis niloticus*)	Y	4,590	8	338	530	8,700	14,000	11,000
4	Common carp (*Cyprinus carpio*)	Y	4,412	8	483	951	4,600	9,100	6,900
5	Torpedo-shaped catfishes nei (*Clarias* spp) MS	N/Y	1,260	2	125	854	1,500	10,000	5,800
6	Freshwater fishes nei (Osteichthyes) MS		2,515	4	*361*	*724*	3,500	7,000	5,200
7	Silver carp (*Hypophthalmichthys molitrix*)		4,828	9	700	1,500	3,200	6,900	5,100
8	Milkfish (*Chanos chanos*)		1,537	3	250	425	3,600	6,100	4,900
9	Yellow catfish (*Pelteobagrus fulvidraco*)		537	1	75	250	2,100	7,200	4,700
10	Grass carp (*Ctenopharyngodon idellus*)		5,728	10	1,000	4,535	1,300	5,700	3,500
11	Silver barb (*Barbonymus gonionotus*)		420	1	100	200	2,100	4,200	3,200
12	Bighead carp (*Hypophthalmichthys nobilis*)		3,146	6	750	2,000	1,600	4,200	2,900
13	Tilapias nei (*Oreochromis(=Tilapia)* spp) MS	N/Y	1,100	2	300	530	2,100	3,700	2,900
14	Catla (*Catla catla*)		3,286	6	1,000	2,000	1,600	3,300	2,500
15	Striped catfish (*Pangasianodon hypophthalmus*)		2,682	5	933	1,385	1,900	2,900	2,400
16	Asian swamp eel (*Monopterus albus*)		314	1	100	200	1,600	3,100	2,400
17	Roho labeo (*Labeo rohita*)		1,993	4	863	2,000	1,000	2,300	1,700
18	Wuchang bream (*Megalobrama amblycephala*)		763	1	450	500	1,500	1,700	1,600
19	Marine fishes nei (Osteichthyes) MS		752	1	*361*	*724*	1,000	2,100	1,600
20	Japanese eel (*Anguilla japonica*)		266	<1	146	249	1,100	1,800	1,400
21	Amur catfish (*Silurus asotus*)		360	1	250	250	1,400	1,400	1,400
22	Rainbow trout (*Oncorhynchus mykiss*)	Y	917	2	507	896	1,000	1,800	1,400
23	Giant gourami (*Osphronemus goramy*)		206	<1	200	300	690	1,000	860
24	Mrigal carp (*Cirrhinus mrigala*)		524	1	600	700	750	870	810
25	Gilthead seabream (*Sparus aurata*)		259	<1	300	400	650	860	750
26	European seabass (*Dicentrarchus labrax*)	Y	263	<1	301	422	620	870	750
27	Cyprinids nei (Cyprinidae) MS		679	1	*666*	*1,436*	470	1,000	750
28	Largemouth black bass (*Micropterus salmoides*)		480	1	680	680	710	710	710
29	Blue-Nile tilapia hybrid (*O aureus x O niloticus*)		411	1	603	603	680	680	680
30	Mandarin fish (*Siniperca chuatsi*)		337	1	500	500	670	670	670
31	Snakehead (*Channa argus*)		462	1	700	700	660	660	660
32	Large yellow croaker (*Larimichthys croceus*)		226	<1	350	350	640	640	640
33	Climbing perch (*Anabas testudineus*)		59	<1	90	100	590	660	630
34	Channel catfish (*Ictalurus punctatus*)		454	1	695	769	590	650	620
35	Africa-bighead catfish hybrid (*Clarias gariepinus* x *C macrocephalus*)		102	<1	150	350	290	680	490
36	Atlantic salmon (*Salmo salar*)	Y	2,616	5	4,719	6,346	410	550	480
37	Pangas catfishes nei (*Pangasius* spp) MS		531	1	933	1,500	350	570	460
38	North African catfish (*Clarias gariepinus*)	Y	236	<1	423	854	280	560	420
	**Total of above**		**52,365**	93			**75,000**	**160,000**	**120,000**
	Total others	N/Y	3,962	7			3,400	6,700	5,000
	**Total estimate**		**56,327**	100			**78,000**	**170,000**	**120,000**

Species with stunning parameters for automated humane slaughter are indicated. Fish numbers are estimated from aquaculture production tonnages (FAO 2021) and estimated mean fish weights (EMWs). For categories comprising a single species, EMWs are based on corresponding fish size data. For multi-species categories (denoted ‘MS’) representing a genus (scientific name ending ‘spp’), EMWs are usually based on EMWs for related single-species categories. For more diverse categories, and those without fish size data obtained, EMWs are extrapolated (shown in italics). ‘Nei’ means unspecified species ‘not elsewhere included’. Source of species with stunning parameters: Spence (2014 cited in HSA 2018).

### Estimates for multi-species categories

A total estimated number range of 10–33 billion was obtained for 17 multi-species categories with an EMW (Table S4), comprising 11% of total 2019 finfish tonnage. The top four are shown in Table 3. Of these 17 EMWs, two were obtained using the method described for single-species EMWs, using the fish size data obtained for a genus or family.

### Estimates for species categories without an EMW

A total estimated range of 6–12 billion was obtained for species categories without an EMW, based on GEMWs (Table S4). These comprised 8.5% of total 2019 finfish tonnage. Most of this, nearly 8% of total tonnage, comprised diverse species categories without species or genus information, such as ‘Freshwater fishes nei’ or ‘Cyprinids nei’ (Cyprinidae) (Table 3). Less than 1% of total tonnage corresponded to single-species and single-genus categories for which no corresponding fish size data were obtained.

### Estimate totals

The estimate obtained for global farmed fish numbers totalled a range of between 78 and 171 billion, with a mid-point of 124 billion, or 1.24 × 10^11^ (Table 3). More details, including estimates for all 274 species categories and derivation of multi-species EMWs and GEMWs, are given in Table S4. Pond loach (*Misgurnus anguillicaudatus*) is the most numerous species with estimated numbers of 14–36 billion (Table 3).

**Figure 1.**
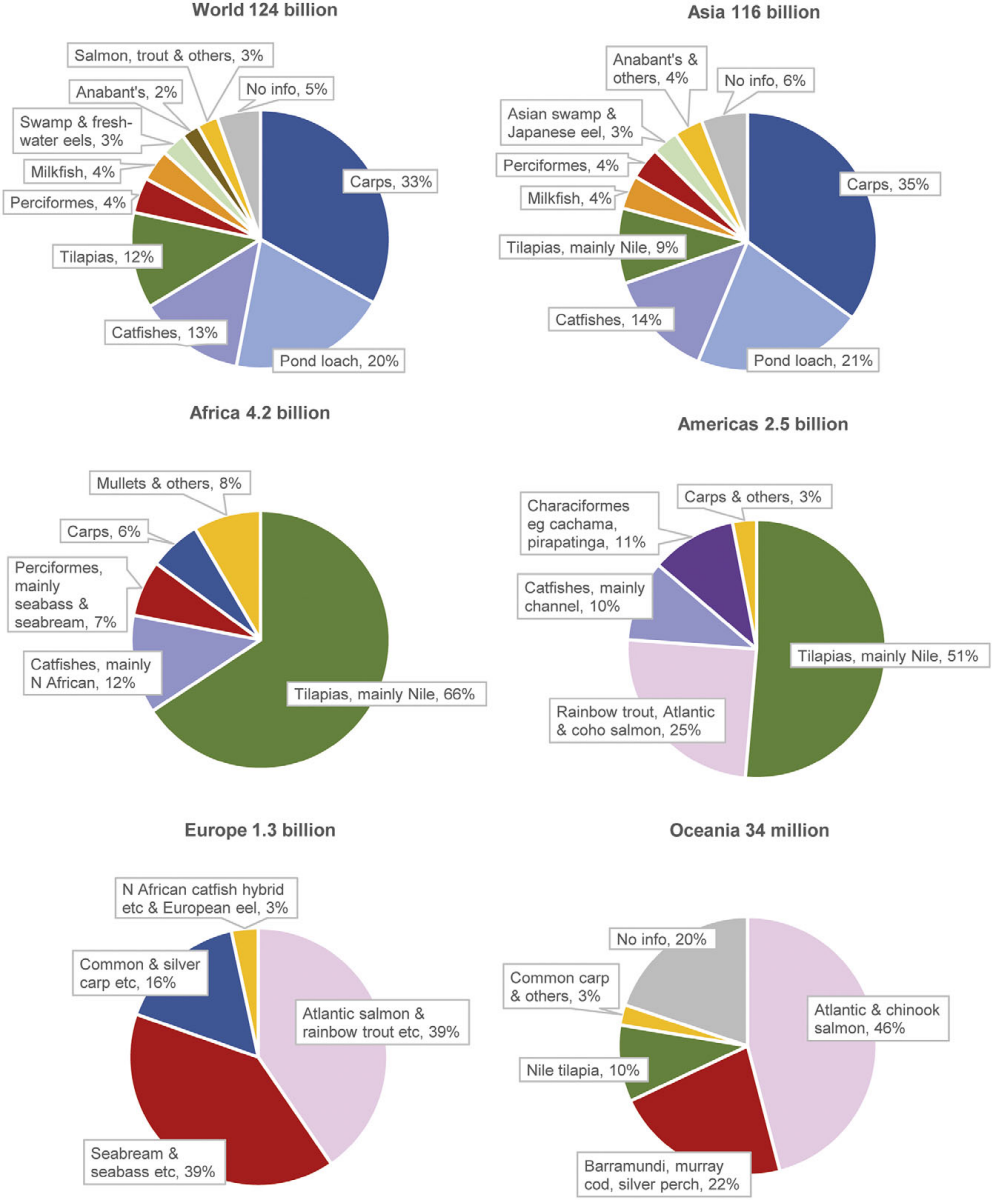
Estimated farmed finfish numbers by species group and continent (2019). These charts show estimated farmed finfish numbers (i.e. mid-points of estimated number ranges) by type of species, globally and in each continent. Anabant’s (Anabantiformes) include gouramies, snakeheads and climbing perch. Perciformes comprise a wide range of species, including mandarin fish in China, European seabass, gilthead seabream, barramundi, murray cod and silver perch. Production is dominated by salmonids and Perciformes in Europe and Oceania; tilapias in Africa and Americas; and cyprinids (carps and pond loach) in Asia and globally.

Estimated numbers (mid-points) are shown by species group and geographical region in Figure 1.

The vast majority of fishes, 94%, are reared in Asia. Carps (cyprinids other than pond loach), pond loach, tilapias (Cichlidae) and catfishes (Siluriformes) together account for 78% of the global estimate.

**Figure 2.**
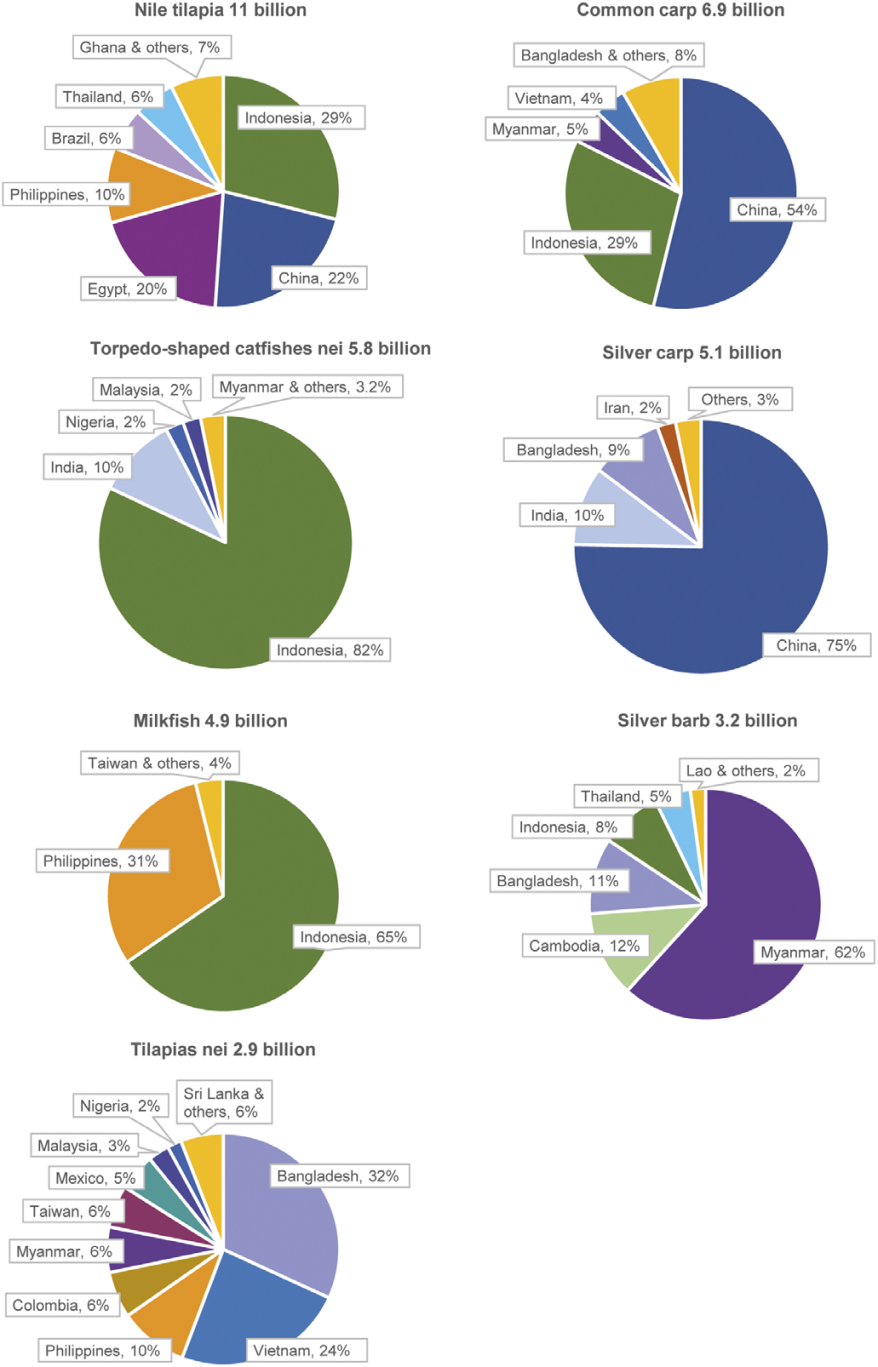
Estimated numbers for a number of top FAO farmed fish species categories, by country (2019). These charts show estimated numbers (i.e. mid-point of estimated number ranges), by country, for seven of the top 13 species categories in FAO finfish aquaculture statistics (Table 3). China and/or Indonesia dominate production numbers for the top five species shown. Top species that are almost entirely farmed in China are not shown (pond loach, carassius carp, yellow catfish, grass and bighead carp).

Estimated numbers for the top species are shown by country in Figure 2, excluding species exclusively or almost exclusively farmed in China. Asian countries dominate production.

Estimated numbers for 28 non-EU countries, and the EU27 countries combined, are shown in Table 4. The top countries, by estimate mid-point, are China, Indonesia and India.

**Table 4.** Estimated numbers of farmed fishes killed for food by country (2019), with comparative birds and mammals and stated legal protection

Rank	Country	Finfish production^1^ ‘000 t	% Total fish tonnage	Estimated numbers (millions)^1^ to 2 sig fig			% Total numbers (midpoint)	Number of birds (millions)^1^	Number of mammals (millions)^1^	Any animal welfare law covering fish slaughter?^2^		Top species
				Lower	Upper	Midpoint				General	Some detail	
1	China	27,086	48%	43,000	100,000	72,000	58%	13,776	1,223	No		Pond loach
2	Indonesia	4,913	9%	9,000	22,000	15,000	12%	4,883	31	Yes	No	Torpedo-shaped catfishes nei
3	India	7,006	12%	5,000	10,000	7,700	6%	2,765	109	Yes	No	Catla
4	Bangladesh	2,343	4%	3,200	5,700	4,500	4%	347	36	No		Tilapias nei
5	Vietnam	3,137	6%	3,000	5,600	4,300	3%	633	46	Yes	No	Striped catfish
6	Philippines	709	1%	2,200	3,800	3,000	2%	1,242	32	Yes^3^	No	Milkfish
7	Myanmar	1,020	2%	1,900	3,900	2,900	2%	1,397	26	No		Silver barb
8	Egypt	1,642	3%	2,100	3,700	2,900	2%	994	46	No		Nile tilapia
9	Thailand	431	1%	740	2,300	1,500	1%	1,287	14	No		Nile tilapia
10	Turkey	367	1%	810	1,400	1,100	1%	1,215	33	Yes	No	European seabass
11	Cambodia	292	1%	620	1,200	930	1%	25	3	?		Silver barb
12	Brazil	530	1%	630	1,200	900	1%	5,906	89	No		Nile tilapia
13	EU27	536	1%	620	1,000	810	1%	6,694	337	Yes^4^	No^4^	Gilthead seabream
14	Iran	450	1%	600	770	690	1%	1,970	8	No		Rainbow trout
15	Nigeria	289	1%	340	830	590	<1%	240	56	Yes	No	North African catfish
16	Taiwan	198	<1%	320	670	490	<1%	412	8	Yes	No	Milkfish
17	Japan	278	<1%	270	530	400	<1%	803	17	No		Silver seabream
18	Malaysia	153	<1%	210	530	370	<1%	630	2	Yes	No	Torpedo-shaped catfishes nei
19	Colombia	165	<1%	220	480	350	<1%	965	11	Yes^5^	No	Tilapias nei
20	South Korea	112	<1%	180	370	280	<1%	1,045	19	No		Bastard halibut
21	USA	203	<1%	250	290	270	<1%	9,594	167	No		Channel catfish
22	Norway	1,451	3%	210	310	260	<1%	70	3	Yes^3^	Yes	Atlantic salmon
23	Peru	57	<1%	210	270	240	<1%	787	65	Yes^3^	No	Rainbow trout
24	Chile	990	2%	190	280	230	<1%	311	7	Yes	No	Atlantic salmon
37	UK	203	<1%	42	59	51	<0.1%	1,136	29	Yes	No	Atlantic salmon
39	Canada	144	<1%	27	53	40	<0.1%	796	52	Some^6^	?	Atlantic salmon
48	Australia	73	<1%	15	36	26	<0.1%	667	47	Some^6^	NSW?^6^	Atlantic salmon
73	Switzerland	2	<0.01%	4.7	7.4	6.1	<0.01%	78	4	Yes^3^	Yes	European perch
82	New Zealand	14	<0.1%	4.1	4.1	4.1	<0.01%	125	27	Yes^5^	Yes	Chinook salmon
	**Total above**	**54,794**	**97%**	**76,000**	**170,000**	**120,000**	98%	**60,793**	**2,548**			
	Others	1,534	3%	1,600	2,800	2,200	2%	15,956	898			
	**World**	**56,327**	100%	**78,000**	**170,000**	**120,000**	100%	**76,748**	**3,445**			

1 Source of fish tonnage: FAO (2021). Source of fish numbers: see text. Source of birds & mammals: FAO (2020a).

2 According to the authors’ interpretation, laws in several countries contain general animal welfare requirements covering farmed fish slaughter, usually one to avoid unnecessary suffering (see Table S8). Law with some detail means fish-specific, but not species-specific, slaughter welfare codes.

3 Law appears specifically to require proper stunning, or that immediate unconsciousness is caused, at slaughter.

4 No detailed law at EU level.

5 Law specifically requires rapid loss of consciousness or rapid death.

6 No federal law. Yes in one or more states but not all.

**Figure 3.**
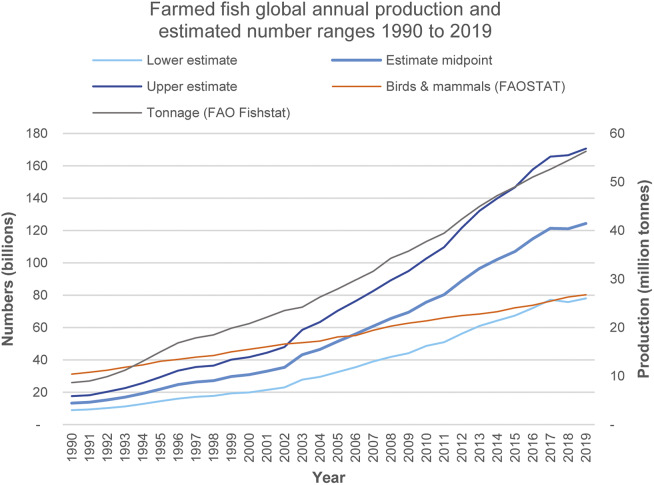
Growth in annual global farmed finfish production since 1990 (FAO 2021). Estimated fish number ranges are shown together with combined numbers of birds and mammals slaughtered (FAO 2020a). According to the estimate mid-points for each year, fish numbers first exceeded bird and mammal numbers in 2006, when they reached 56 billion. By 2019, they had reached 124 billion, representing 1.5 times bird and mammal numbers.

Finfish tonnage and the estimate mid-point have both increased every year between 1990 and 2019 except 2018 (Figure 3). The estimate mid-point exceeds the number of birds and mammals killed for food for all years since 2006 (Figure 3). By 2019, the lower estimate nearly equals the 80 billion terrestrial animals slaughtered for food that year (FAO 2020a). Data for Figure 3, including annual estimates for 1990 to 2019, are given in Table S5 of the supplementary material.

Between 1990 and 2002, tonnage and the estimate mid-point showed a similar pattern of growth, increasing 2.7-fold from 9 to 24 million tonnes and from 13 to 35 billion fishes. Between 2002 and 2019, the respective increases in tonnage and estimate mid-point were 2.4- and 3.5-fold, to 56 million tonnes and 124 billion fishes.

### Sensitivity analysis

Alternative estimates A1–A4 tested the effects of different data ranking systems used in the generation of EMWs (Table 1).

Alternative estimate A1 included all 229 fish sizes and gave a wide range of 66–269 billion. A2, which excluded weights from marketing websites/reports where possible, gave a range of 66–253 billion. A3, which additionally excluded common weights where possible, gave a range of 66–245 billion. A4, which prioritised data for the top producing country and global sizes, gave a much narrower range of 74–169 billion.

The main estimate, which modified A4 by giving still higher priority to data for the country being estimated, gave a slightly increased estimate of 78–171 billion. Differences between the main estimate and A4 are shown in Table S2.

For alternative estimates that assumed weights from marketing websites and reports, which did not say otherwise, were headed and gutted, A5 gave a range of 73–154 billion and A6 gave a range of 70–149 billion.

### Analysis of fish weights obtained from a census

Census data obtained in the present study included the Scottish fish farm survey (MSS 2020) and US aquaculture censuses (USDA 2007, 2014, 2019).

The first census data comparisons analysed fish slaughter weights from these sources for change over time. Annual mean slaughter weights for Scottish Atlantic salmon (*Salmo salar*) increased from 4.1 to 5.2 kg between 2002 and 2019 (Table S6 of the supplementary material). An estimate of Scottish salmon numbers in 2019 based on the mean weight in 2002 would therefore have over-estimated numbers by 28%.

Mean slaughter weights for six US farmed fish species (Table S7 of the supplementary material), between 2005 and 2018, each changed by between –40% and 35%, such that an estimate of numbers in 2018, based on their mean weights in 2005, would have over-estimated numbers by around 10%.

The second census data comparisons compared fish size data from these censuses with data from other sources, where both were obtained for a species in a country. Three species had such fish weight data, enabling comparison between them (Table 5). Weight ranges for two of these, Atlantic salmon and rainbow trout (*Oncorhynchus mykiss*), were similar. However, census data doubled the lower weight for channel catfish (*Ictalurus punctatus*), so halving its upper estimated numbers.

**Table 5.** Comparison of estimated farmed fish numbers based on census and other data

Species and census	Mean slaughter weight (g)	Estimated numbers 2019 (millions)
Channel catfish US:		
non census data	340–680	226–451
census data^1^	695–769	199–221
Difference		–26 to –231
Percent difference		–12% to –51%
Rainbow trout US:		
non-census data	450–600	26–34
census data^1^	488–625	25–31
Difference		–1 to –3
Percent difference		–4% to –8%
Atlantic salmon UK:		
non-census data	4,500–5,500	35–42
census data^2^	4,113–5,366	36–46
Difference		1 to 4
Percent difference		3% to 9%

For rainbow trout and Atlantic salmon, estimates were similar. For channel catfish, non census data halved the lower EMW and doubled the upper estimate.

1 USDA 2007, 2014, 2019). Converted from pounds.

2 MSS (2020) annual mean weight 2002–2019.

### Slaughter analysis

Stunning parameters are currently published for six of the top 31 single-species categories (Table 3), according to Spence (2014; cited in HSA 2018). These include parameters for in-water electrical, dry electrical and percussive stunning. Parameters of all three types are published for common carp (*Cyprinus carpio*), rainbow trout, Atlantic salmon and North African catfish (*Clarias gariepinus*); while those published for Nile tilapia and European seabass (*Dicentrarchus labrax*) relate to in-water stunning.

Estimated numbers for all fishes for which species-specific stunning parameters are currently published, according to Spence (2014; cited in HSA 2018), totalled 16–40 billion in 2019 or, respectively, 20 and 24% of the lower and upper total estimate. This includes 16–27 billion for the six single-species categories listed above, and up to 14 billion for multi-species categories ‘Tilapias nei’ (*Oreochromis* spp) and ‘Torpedo-shaped catfishes nei’ (*Clarias* spp), which are likely to include many Nile tilapia and North African catfish.

At the time of writing, only percussive stunning parameters are published for gilthead seabream (*Sparus aurata*); this is not included in the above estimate since percussive stunning is not considered suitable for the scale of a typical slaughter operation for this species (HSA 2018).

Many of the top producing countries have some general animal welfare law that, in principle, covers farmed fishes at slaughter but very few have fish-specific welfare requirements (Table 4). The key results from the analysis of law (Table S8 of the supplementary material) are that:

- At least ten countries have no such legal protection, accounting for 70% of the total estimate by mid-point;
- In at least 15 countries, including the UK and together with the EU27, fishes have some stated general protection, either specifically or as vertebrates or living creatures, which is usually a requirement not to cause unnecessary suffering. These account for 28% of the total estimate by mid-point.
- Of these 15, three countries have fish-specific slaughter codes that require stunning and/or prohibit certain inhumane slaughter methods. These three countries account for 0.2% of the total estimate by mid-point.
- Countries that were not analysed accounted for 3% of the total estimate by mid-point.

Certification schemes also have the potential to improve welfare during slaughter. It was estimated that between 1.8 and 2.7 billion finfishes, killed for food annually between 2013 and 2015, were reared on farms within the main global certification schemes (Table S9 of the supplementary material). This represents 2% of the total 2015 estimate of 67–147 billion by mid-point. This includes, to 2 significant figures, 450 million in Global Aquaculture Alliance Best Aquaculture Practices (GAA BAP) and 15 million in organic schemes in 2013; 340 million in Friend of the Sea (FOS) in 2014; 920 million in GLOBALGAP and 560 million in the Aquaculture Stewardship Council (ASC) in 2015. Certified finfish species include ‘tilapia’, Atlantic salmon, European seabass, rainbow trout, ‘pangasius’ and gilthead seabream. Atlantic salmon/‘salmon’ had, by far, the largest certified production tonnage of all species, while ‘tilapia’ had the largest estimated certified fish numbers. The estimate assumes that certified farms are included in one scheme only.

## Discussion

The present study has estimated that 78–171 billion (or 7.8 × 10^10^ to 1.71 × 10^11^) farmed finfishes were killed for food in 2019 (Table 3), exceeding the combined number of farmed birds and mammals (80 billion) killed for food that year (FAO 2020a). Fish numbers have grown dramatically since 1990 (Figure 3), with production increases in all five continents (Table 2), each favouring different species groups (Figure 1). The FAO predicts further increases in farmed fish production (2020b).

According to the present study, estimated fish numbers slaughtered in 2010 totalled 49–103 billion (Table S5), which is a narrower range than the earlier estimate of 37–122 billion for the same year (Mood & Brooke 2012). Compared to the earlier estimate, the estimate for the pond loach is much greater, while the combined estimate for all other species is narrower, mainly due to the new data ranking system.

Here, it is estimated that farmed fish numbers slaughtered in 2018 totalled 76–167 billion (Table S5), including 14–36 billion for the pond loach. This total has the same order of magnitude as the estimated 59–129 billion farmed aquatic vertebrates slaughtered in the same year based on maximum weights (Franks *et al.* 2021), which included a lower figure of 2.5–4.1 billion for the pond loach.

The present study’s fish numbers are best estimates, calculated from the widely accepted FAO aquaculture statistics, together with fish ‘harvest’ or market weight data from various sources. FAO aquaculture statistics are themselves estimates and, while the FAO has actively assured quality as far as possible, the following issues can result in some inaccuracies:

- Countries not sending their statistics on time, whereby prior year data may be used (FAO 2020b);
- Incorrect conversion of processed weights into live weight equivalents (Garibaldi 2012);
- Small-scale fisheries are often under-reported (Bjorndal *et al.* 2014);
- Over-reporting in some countries. China has made two major downward revisions in the last 20 years (FAO 2020b);
- Confusion with capture fisheries; incorrectly including wild fishes caught from stocked public waterbodies (Welcomme 2011).

Some accuracy issues in EMWs, and farmed fishes excluded from the estimate because they are not slaughtered for food, are discussed below.

### Data quality issues associated with EMWs

Since the FAO does not publish mean weights in its aquaculture production statistics, and since there are limited census data giving fish slaughter weights, this estimate is largely based on data from other sources. More census data would be needed to draw any clear conclusion on the likely accuracy of the non-census data.

There is some risk that numbers for some species are over-estimated due to being based on slaughter weights that were assumed to be whole fishes but not stated as such. The estimated maximum effect of correcting for these assumptions, if they were all incorrect, would be to reduce the estimate mid-point by 9–12% (A5 and A6).

The potential variability of fish size between place and time, for a species, increases the difficulty in estimating the global mean weight. As an example of variability between rearing systems, Nile tilapia are reared to 200–500 g and 500–1,000 g for pond and cage culture, respectively, in Thailand (Bhujel 2013). Also, some species are reared to both a portion size and a larger fillet size, such as rainbow trout and turbot (*Psetta maxima*). Scottish Atlantic salmon are slaughtered in three age groups: less than a year, 1–2 years and 2–3 years, for which the range of average slaughter weights between years were, respectively, 1.7–3.5, 3.8–5.1 and 4.4–6.3 kg for smolts put to sea between 2000 and 2017 (MSS 2020).

Estimates for 2003 to 2019 showed numbers increasing faster than tonnage (Table S5), due to changes in the proportions of different species in FAO tonnages, including increased numbers of pond loach and some other small species such as yellow catfish and Asian swamp eel (*Monopterus albus*) (these three first separately reported for China, the main producer, from 2003). Estimates for 1990 to 2019 do not allow for any change in mean weights between years for a given species and country, since they are all based on the same fish size data.

However, Scottish and US census data provide some evidence that mean fish slaughter weights of individual species may increase over time. The mean weight for Scottish farmed Atlantic salmon increased by 28% between 2002 and 2019 (Table S6), while the mean weight for six species combined in the US increased by 10% between 2005 and 2018 (Table S7). If this pattern occurs for other species globally, then numbers may be over-estimated for those species.

We recommend that the FAO provides estimates of numbers of fishes slaughtered, alongside production tonnages, to facilitate animal welfare assessment. The law in most of the top 24 producing countries (counting the EU27 as one country for this purpose since it has common legislation) recognises, at some level, a need to protect the welfare of farmed fishes (Table 4) and the FAO already collects data for hatchery production in numbers (FAO 2020c).

### Fishes excluded from the estimates

These estimates include only finfishes recorded in FAO aquaculture statistics, which include only those designated for food (FAO 2018, 2020c). They exclude the following:

- Farmed fishes that die during rearing (‘mortality’);
- Cleaner’ fishes used on salmon farms to eat and control lice;
- Fishes killed, but not used for food, when ponds are drained;
- Fishes reared for bait;
- Fishes reared to feed live to carnivorous fishes, e.g. mandarin fish (*Siniperca chuatsi*);
- Fishes reared for release into the wild (stock enhancement);
- Fishes reared for ‘ornamental purposes’;
- Wild-caught fishes including those caught for aquaculture feed;
- Other farmed and wild-caught species, e.g. decapods.

The scale of farmed fish mortality during rearing is largely unknown as mortality levels are usually not published (to the best of the authors’ knowledge). However, annual mortality rates for Atlantic salmon during the marine stage of 14.2–16.2% in Norway for 2015–2019 (NVI 2020), and 13–28% in Chile for 2015–2017 (Aquabench 2021), have been reported. In Scotland, the mortality for each cohort of Atlantic salmon smolts first put out to sea in a given year between 2012 and 2017 totalled 14.6–26.7% (MSS 2020). For Nile tilapia, Asian and African case studies suggest higher mortality rates, ranging between 30 and 50% during the grow-out phase (Rana & Hasan 2013). Lower mortality rates of 5% for crucian carp (*Carassius carassius*) and silver carp (*Hypophthalmichthys molitrix*) are reported for some large Chinese farms (Yuan Xihe 2016). Prior to the grow-out phase, mortality rates may be higher. In Norway, approximately 25% of hatched salmon fry never reach the sea phase (NVI 2019). Some reported mortality rates, for the top 12 species, are shown in Table S10 of the supplementary material.

Regarding cleaner fishes, 49.1 million were placed in sea cages in Norway in 2019, including farmed lumpfish (*Cyclopterus lumpus*) and mostly wild-caught wrasse species (Labridae) (NVI 2020). A Norwegian study of cleaner fishes in salmon cages recorded mortalities of 7.2 and 9.9% in ballan wrasse (*Labrus bergylta*) and lumpfish, respectively, over a four-month period (Geitung *et al.* 2020). Total losses, including escapes, were 57% for ballan wrasse and 27% for lumpfish. Scottish aquaculture reared 660,000 lumpfish and 59,000 wrasse cleaner fishes in 2019, comprising, respectively, 13 and 3 tonnes (MSS 2020). Numbers of wild-caught wrasse used on Scottish fish farms are likely to be far higher, with UK wild wrasse capture totalling 68.8 tonnes in 2016, mostly landed live with comparatively few sold for consumption (Riley *et al.* 2017).

Ponds are often drained to kill the fish for food and/or to kill any unwanted fry and other fish species at the end of a production cycle, e.g. tilapia ponds may be drained or treated with pesticides to kill the fry (FAO 2022b).

Fishes farmed for bait and live feed may comprise large numbers. For example, US fish farms sold 1.2 billion bait fishes with an average weight of 3 g in 2018 (USDA 2019). Welfare of bait fish may be extremely poor. For example, farmed milkfish (*Chanos chanos*) fingerlings are sometimes used as live bait in long-line tuna fishing (ICAR-CIBA 2016).

The FAO (2022c) describes a system in which 4,500 hatchling carp are concurrently stocked to feed each mandarin fish. If this is typical then mandarin farming overall consumes an estimated 3,000 billion feed fishes, i.e. 3.0 × 10^12^, based on an estimated 674 million mandarin fish (Table 3).

The release of young fish from hatcheries into the wild to enhance, or restore, the population often results in high death rates (Braithwaite & Salvanes 2010). China released 109 billion young fishes, apparently, over 2006–2011 (Chinese MOA 2014), and the USA released 3.5 billion in 2018 (USDA 2019).

Most commercially caught wild fish are not intentionally slaughtered, but die as a consequence of capture or processing, and are frequently gutted alive (Metcalfe 2009). In general, they are not stunned for unconsciousness prior to killing (Breen *et al.* 2020). An estimated 0.97–2.7 trillion fishes (0.97–2.7 × 10^12^) were caught in recorded capture each year between 1999 and 2007 (Mood & Brooke 2010) (not peer-reviewed). This estimate was updated to 0.79–2.3 trillion for 2007–2016 (Mood & Brooke 2019a). These estimates exclude bycatch mortalities and other unrecorded capture.

Aquaculture consumes large numbers of wild fishes caught and reduced to fishmeal and fish oil. An estimated 0.46–1.1 trillion fishes (0.46–1.1 × 10^12^) were caught for reduction globally each year between 2007 to 2016 (Mood & Brooke 2019b) (not peer-reviewed). Respectively, 70 and 73% of fishmeal and fish oil are used for aquaculture feeds (Mallison 2017), though their inclusion rates (as a percentage of feed) show a clear downward trend (FAO 2020b). Herbivores and omnivores are less dependent on dietary fishmeal and fish oil than carnivorous species, which include most salmonids and marine fish species (Tacon & Metian 2015), although one company reports it has produced a formulated salmon feed that utilises algae oil and contains no fishmeal or fish oil (Skretting 2022).

If efforts to develop mesopelagic fishing succeed, catching finfishes such as lanternfish (e.g. *Benthosema glaciale*) weighing only a few grams (Irigoien *et al.* 2014) from a total estimated biomass of 2–19.5 billion tonnes (Sobradillo *et al.* 2019), then fish numbers caught to make fish feed could substantially increase.

An estimated 5–15 billion farmed crabs (Brachyura); 37–60 billion farmed crayfish and lobsters (Reptantia) and 213–530 billion farmed shrimps and prawns (Natantia) were killed for food globally in 2017 (Mood & Brooke 2019c) (not peer-reviewed). Decapod crustaceans are included in some national animal protection laws (Table S8).

### Animal welfare implications

The number of animals affected is one important measure of any welfare issue and can help identify priorities for research and policy efforts. This estimate indicates that very large numbers of animals would benefit from improved fish welfare during slaughter, transport and rearing.

### Welfare at slaughter for the most numerous farmed fish species

Slaughter methods used on the top species (Table 3) include the following, performed without prior stunning and therefore not in accordance with OIE guidelines for protecting fish welfare during slaughter (see *Introduction*):

- Salt bath: pond loach (Gibson *et al.* 2020);
- Asphyxiation in air: Nile tilapia (Pedrazzani *et al.* 2020);
- Asphyxiation in ice or ice slurry: silver carp (Zhang *et al.* 2017), Nile tilapia (Pedrazzani *et al.* 2020), milkfish (FAO 2022d);
- Exsanguination (gill-cutting): silver carp (Zhang *et al.* 2017), ‘tilapia’ (Lines & Spence 2012), striped catfish (Sørensen 2005), ‘pangasius’ (King-Nobles *et al.* 2020);
- Evisceration (gutting): ‘tilapia’ (Lines & Spence 2012);
- Decapitation: pond loach (Gibson *et al.* 2020), ‘pangasius’ (Lines & Spence 2012).

Note that pangasius species, of which striped catfish is the most commonly farmed species (ASC 2022a), are commonly referred to simply as ‘pangasius.’

Nile tilapia (FAO 2022b), crucian carp (FAO 2022e) (a *Carassius* species), common carp (FAO 2022f) and silver carp (FAO 2022g) are often sold live to the consumer to kill at home, including in Europe (EU Commission 2017). This may involve asphyxia, temperature shock, excessive handling and ineffective stunning (EFSA 2009).

Parameters for humane stunning, with stunning machines, have been published for six of the top 38 species (Table 3), highlighting the need for more research. For the species that do have stunning parameters, slaughter methods may only stun the majority but not all fishes, and therefore require ongoing improvement (HSA 2018).

### Analysis of fish welfare law

Although many countries have a general legal requirement to avoid unnecessary suffering during slaughter that covers fish species, only a few have fish-specific welfare requirements for farmed fish slaughter; including Norway, Switzerland and New Zealand (Table 4 and Table S8). Norwegian law requires farmed fishes to be stunned before slaughter but permits live cooling with CO_2_ (Norwegian Government 2018), which does not meet OIE guidelines, though this method is being phased out (European Commission 2017). Swiss law states that fishes, farmed or wild-caught, must be stunned prior to killing (Swiss Federal Council 2018). However, alongside percussion, electrical stunning and spiking; its list of permitted stunning methods includes cervical dislocation, which is not a method recommended by OIE guidelines. New Zealand law on farmed fish slaughter (New Zealand Government 2022), which also covers wild-caught fishes killed in restaurants, prohibits gill-cutting and desliming of eels without prior stunning. However, the law appears to include contradictory clauses; although it requires a ‘rapid’ loss of consciousness, it also appears to permit chilling followed by asphyxiation in air without stunning. So far as the authors can determine, none of the countries analysed currently has species-specific prescribed methods or stunning parameters for fish slaughter.

Although EU law (Regulation [EC] No 1099/2009, which has also transferred into UK law) prohibits causing fishes avoidable pain or suffering during slaughter (European Union 2009), a study comparing slaughter practices with OIE guidelines in some EU28 countries and Norway revealed widespread non-adherence for five farmed species studied (European Commission 2017). Slaughter of Atlantic salmon with prior percussive stunning was found to be adherent in Norway, the UK and the Republic of Ireland. However, the study found that the following non-adherent and stressful methods are still being used in Europe:

- CO_2_ stunning of Atlantic salmon (the Republic of Ireland) and rainbow trout (France);
- Chilling with CO_2_ of Atlantic salmon (Norway);
- Air-exposure of common carp for 10 min prior to percussive stun (Poland);
- Asphyxia in ice or ice slurry of rainbow trout (Denmark and Poland), European seabass and gilthead seabream (in Greece, Spain, Italy and Turkey);
- Chilling in ice water followed by electrical stunning of rainbow trout (France);
- Electrical stunning of Atlantic salmon without decapitation (Norway).

This study was also unable to determine whether electrical stunning equipment used for rainbow trout and common carp in the countries surveyed was effective (European Commission 2017). These findings show that general statements to minimise suffering cannot be relied upon to ensure adherence to OIE guidelines, and the need for detailed species-specific legislation to ensure welfare during slaughter, with stunning parameters where available. Effective enforcement is also required.

### Voluntary farmed fish welfare codes

Certification scheme standards, and the requirements of food businesses, have great potential for improving the welfare of large numbers of farmed fishes. Certified fish numbers, here estimated as 1.8–2.7 billion for 2013–2015 (Table S9), are likely to have increased since then. It is reported that total annual certified tonnage (including crustaceans) for GAA BAP grew to 1.5 million by the end of 2018 (The Fish Site 2019), which is more than double the 0.71 million tonnes it certified in 2013 (Potts *et al.* 2016). Note that the GAA BAP scheme is now known as Global Seafood Alliance Best Aquaculture Practices (GSA BAP).

Global aquaculture certification schemes FOS (2021), GLOBALGAP (2021), GSA BAP (2016, 2020, 2021), and the International Federation of Organic Agriculture Movements (IFOAM 2019) and Naturland (2022) organic standards, all currently have some fish welfare rules, including some for slaughter. Of these, FOS (2021) and Naturland (2022) standards, and GSA BAP (2016) standards for salmon species, have species-specific requirements which include stocking density limits. Although geographically limited to the UK, it is noted that the RSPCA Assured scheme is welfare-specific and has detailed species-specific standards for farmed Atlantic salmon and rainbow trout (RSPCA 2020, 2021), including stocking density limits and requirements for percussive or electrical stunning.

FOS (2021) farmed fish welfare standards will require pre-slaughter percussive or electrical stunning from November 2024. These other global standards require percussive or electrical stunning, at least in some cases. IFOAM (2019) standards require aquatic vertebrates to be stunned before killing. GSA BAP standards require that farmed fishes are slaughtered by a humane method (GSA BAP 2021); or that, before slaughter, they are humanely stunned instantly (GSA BAP 2016) or humanely and quickly rendered unconscious (GSA BAP 2020). However, GSA BAP (2021) currently still permits rapid chilling in an ice-bath for on-farm killing of tropical fish species and certain fish types, as an exception to its humane slaughter requirement. Naturland (2022) requires pre-slaughter stunning of fishes by concussion, electrocution or plant anaesthetics; but currently still permits use of ice, without pre-stunning, for tropical and subtropical fish species. GLOBALGAP (2021) requires fish to be stunned using an effective stunning method and to immediately become unconscious and that, ‘where effective automation technology is available’, percussive and/or electro-stunning is employed. At the time of writing, global certification scheme ASC has released a draft fish welfare standard (ASC 2022b). If this is adopted in its current form, ASC certified farms would be required to stun fish with percussive or electrical methods following a transition period (0–6 years from April 2025 depending on the species).

Some retailers’ own policies, at least in the UK, require pre-slaughter stunning for branded and/or own-label farmed fishes, according to their publications, e.g. Tesco (2021) and Sainsbury’s (2021).

Increasing the numbers of fishes certified, with improved species-specific welfare standards, could improve the welfare of billions of farmed fishes.

## Conclusion

Our estimate of 78–171 billion farmed fishes slaughtered for food in 2019, in addition to others affected including on-farm mortalities and those reared or captured for feed, demonstrates the very large number of fishes whose welfare is affected by aquaculture practices. We recommend the FAO collects and publishes statistics for farmed fish production in numbers, as well as tonnages, to facilitate animal welfare assessment. Our study also establishes the considerable potential for improving welfare of farmed fishes through detailed fish- and species-specific legislation, voluntary standards and their enforcement; further research and development; and industry practice.

## Supplementary material

For supplementary material accompanying this paper visit https://doi.org/10.1017/awf.2023.4. click here to view supplementary material
