# Supplementary material for: Estimating global numbers of farmed fishes killed for food annually from 1990 to 2019
Source: Anim Welf. 2023 Feb 6;32:e12. doi: 10.1017/awf.2023.4 (PMC10936281; doi:10.1017/awf.2023.4)
Supplement: Supplementary file 1 [file S0962728623000040sup001.zip › S0962728623000040supp001.pdf]

Table S1 Types of fish size references used to estimate global mean weights for farmed fish species

Reference type		No. of reported farmed fish sizes obtained (eg harvest or market)						Total no. fish sizes	Total no. reference docs
		Average or mean weight	Simple weight range	Usual or most common weight	Preferred or optimal weight	Common or typical weight	Weight from marketing website/ report		
1	Research articles	7	16	15	1	1		40	37
2	FAO cultured species information webpages	4	12	12	5	1		34	27
3	Other FAO reports	6	15	8			1	30	24
4	Government and inter-government (except FAO)	25	2	1				28	10
5	Books about aquaculture and farming		12	10	1			23	11
6	Industry factsheets etc, trade journals	1	8	3	2			14	13
7	Research institute and university documents		5	5				10	8
8	NGO reports and factsheets	2	7	1				10	9
9	Misc websites concerning fish farming	1		2		1		4	4
10	Marketing website						2	2	2
11	Patent for pond polyculture method		1					1	1
	**Total fish sizes obtained**	**46**	**78**	**57**	**9**	**3**	**3**	**196**	**146**
		**23%**	**40%**	**29%**	**5%**	**2%**	**2%**	**100%**	
	By country type:								
	Global	4	13	10	4			31	**16%**
	Top producing country	16	22	25			1	64	**33%**
	Other countries	26	43	22	5	3	2	101	**52%**
	**Total by country type**	**46**	**78**	**57**	**9**	**3**	**3**	**196**	**100%**
