# Supplementary material for: Estimating global numbers of farmed fishes killed for food annually from 1990 to 2019
Source: Anim Welf. 2023 Feb 6;32:e12. doi: 10.1017/awf.2023.4 (PMC10936281; doi:10.1017/awf.2023.4)
Supplement: Supplementary file 1 [file S0962728623000040sup001.zip › S0962728623000040supp002.pdf]

Table S2 Summary of differences between estimate A4 and the main estimate

This table shows the effect of including estimates for individual countries in main estimate.

FAO species category	Producing country (top producing country in bold)	FAO prod'n 2019 000 (t)[1]	Estimate A4					Main estimate					Difference in estimated numbers (millions) rounded	
			Size type EMW based on[2]	EMW		Estimated number range (millions)		Size type EMW based on[2]	EMW for country (g). Global mean weight in italics		Adjusted estimated number range (millions)			
				Lower	Upper	Lower	Upper		Lower	Upper	Lower	Upper	Lower	Upper
Silver carp	Global except below	4,596	1	750	1,500	3,218	6,437	2	750	1,500	3,064	6,128		
	Bangladesh	232						2	300		154	772		
	Total	**4,828**				**3,218**	**6,437**		*700*	*1,500*	**3,218**	**6,900**		**463**
Nile tilapia	**China**	1,231	1,3	400	662	6,934	11,476	2,1	400	662	1,860	3,078		
	Indonesia	1,185						1	369	369	3,211	3,211		
	Thailand	214						2	200	1,000	214	1,069		
	Philippines	174						2,3	100	300	581	1,742		
	Ghana	46						2	250	350	131	183		
	Uganda	71						3	700	700	102	102		
	Zambia	28						3	350	350	81	81		
	Other countries	1,641						2,1	400	662	2,479	4,103		
	Total	**4,590**				**6,934**	**11,476**		*338*	*530*	**8,657**	**13,568**	**1,723**	**2,092**
Common carp	**China**	2,885	2	650	969	4,554	6,788	3	650	969	2,978	4,439		
	Indonesia	631						2	200	800	789	3,156		
	Europe	168						2	1,000	2,500	67	168		
	Vietnam	134						2	300	800	168	448		
	India	25						3	500	500	49	49		
	Japan	3						2	250	800	3	11		
	Other countries	565						3	650	969	584	870		
	Total	**4,412**				**4,554**	**6,788**		*483*	*951*	**4,639**	**9,141**	**85**	**2,353**
Striped catfish	**Vietnam**	1,600	1	1,000	1,500	1,788	2,682	2,3	1,000	1,500	1,067	1,600		
	Bangladesh	447						2	700	1,000	447	639		
	Global except above	635						2	1,000	1,500	423	635		
	Total	**2,682**				**1,788**	**2,682**		*933*	*1,385*	**1,937**	**2,874**	**149**	**192**
Roho labeo	Global except below	737	1,2	700	2,000	996	2,847	2	700	2,000	368	1,053		
	**India**	1,256						2,3	1,000	2,000	628	1,256		
	Total	**1,993**				**996**	**2,847**		*863*	*2000*	**996**	**2,309**		**-538**
Milkfish	Global except below	1,127	1,2	250	500	3,074	6,148	3	250	500	2,254	4,508		
	Philippines	410						2	250	300	1,366	1,640		
	Total	**1,537**				**3,074**	**6,148**		*250*	*425*	**3,620**	**6,148**	**547**	

Rainbow trout	Global except below	61						2	280	5,000	12	219		
	Europe except below	224						4	1,000	2,000	112	224		
	**Iran**	206						3	500	500	412	412		
	Chile, Norway, Finland, Sweden	188						4	3,000	5,000	38	63		
	Turkey	123	1	280	5,000	183	3,273	1	210	583	211	586		
	Peru	51						2	200	250	203	254		
	China	39						2	3,000	5,000	8	13		
	USA	15						1	488	625	25	31		
	Canada	9						1,4	1,200	5,000	2	7		
	Total	**917**				**183**	**3,273**		*507*	*896*	**1,022**	**1,809**	**839**	**-1,464**
Blue-Nile tilapia, hybrid	**China**	411	3	220	603	681	1,866	1	603	603	681	681		**-1,185**
	Global except above	<1						1	220	603	<1	<1		
	Total	**411**				**681**	**1,866**		*603*	*603*	**681**	**681**		
Total above		**21,369**				**21,429**	**41,517**				**24,772**	**43,430**	**3,342**	**1,913**
Others		**9,969**				**4,802**	**12,169**				**5,065**	**12,062**	**263**	**-107**
**Total above**		**31,338**				**26,231**	**53,686**				**29,837**	**55,492**	**3,606**	**1,806**
Species with no differences		14,046				32,819	70,113				32,819	70,113		
**Total**		**45,384**				**59,050**	**123,799**				**62,656**	**125,605**	**3,606**	**1,806**

1 FAO (2021).

2 Size types: 1 = average or mean weight; 2 = simple weight range; 3 = usual or most common weight; and 4 = preferred or optimal weight.
