# Supplementary material for: Estimating global numbers of farmed fishes killed for food annually from 1990 to 2019
Source: Anim Welf. 2023 Feb 6;32:e12. doi: 10.1017/awf.2023.4 (PMC10936281; doi:10.1017/awf.2023.4)
Supplement: Supplementary file 1 [file S0962728623000040sup001.zip › S0962728623000040supp003.pdf]

Table S3 Estimated fish numbers for single-species EMWs (2019) by data ranking

Data rank -ing	Geographic region of data	Size type	Prod'n '000 (t)[1]	% Total fish t	Estimated numbers (billions)		% Total estimated numbers	
					Lower	Upper	Lower	Upper
1	Country being estimated	Average, usual, preferred or optimal weights or simple weight ranges	27,908	50%	42	88	54%	51%
	Continent of country being estimated	As above	393	1%	<1	<1	<1%	<1%
2	Global or primary country	As above	14,228	25%	13	24	16%	14%
3	Non-primary country	As above	2,784	5%	8	13	10%	7%
4	Any country	Common weights	68	<1%	<1	<1	<1%	<1%
5	Any country	Marketing weights	4	<1%	<1	<1	<1%	<1%
Total estimate for single-species EMWs:			45,384	81%	63	126	80%	74%
Total estimate			56,327	100%	78	171	100%	100%

1 FAO (2021).
