# Supplementary material for: Estimating global numbers of farmed fishes killed for food annually from 1990 to 2019
Source: Anim Welf. 2023 Feb 6;32:e12. doi: 10.1017/awf.2023.4 (PMC10936281; doi:10.1017/awf.2023.4)
Supplement: Supplementary file 1 [file S0962728623000040sup001.zip › S0962728623000040supp004.pdf]

Estimated global numbers of farmed finfishes killed for food (2019)

Section 1 Summary

Estimate part	Production (t)	Percent of total fish production %	Estimated numbers (millions) lower	Estimated numbers (millions) upper	Estimated numbers (millions) midpoint	
Single-species categories with an EMW:	45,384,315	81%	62,656	125,605	94,130	-
Multi-species categories with an EMW:	6,129,852	11%	9,565	33,490	21,528	-
	**51,514,166**	91%	**72,221**	**159,095**	**115,658**	-
Species without an EMW	4,812,913	9%	*5,845*	*11,651*	8,748	-
**Total**	**56,327,079**	100%	**78,066**	**170,747**	**124,406**	-
			-	-	-	

Section 2 Single-species categories with an EMW

Species	Production (t)	Lower global EMW	Upper global EMW	Numbers lower (millions)	Numbers upper (millions)	Numbers midpoint (millions)
Pond loach (Misgurnus anguillicaudatus)	357,806	10	26	13,605	35,781	24,693
Nile tilapia (Oreochromis niloticus)	4,590,292	338	530	8,657	13,568	11,113
Common carp (Cyprinus carpio)	4,411,900	483	951	4,639	9,141	6,890
Silver carp (Hypophthalmichthys molitrix)	4,827,720	700	1,500	3,218	6,900	5,059
Milkfish (Chanos chanos)	1,536,970	250	425	3,620	6,148	4,884
Yellow catfish (Pelteobagrus fulvidraco)	536,964	75	250	2,148	7,160	4,654
Grass carp(=White amur) (Ctenopharyngodon idellus)	5,728,383	1,000	4,535	1,263	5,728	3,496
Silver barb (Barbonymus gonionotus)	420,342	100	200	2,102	4,203	3,153
Bighead carp (Hypophthalmichthys nobilis)	3,145,900	750	2,000	1,573	4,195	2,884
Catla (Catla catla)	3,286,274	1,000	2,000	1,643	3,286	2,465
Striped catfish (Pangasianodon hypophthalmus)	2,682,246	933	1,385	1,937	2,874	2,406
Asian swamp eel (Monopterus albus)	313,872	100	200	1,569	3,139	2,354
Roho labeo (Labeo rohita)	1,992,876	863	2,000	996	2,309	1,653
Wuchang bream (Megalobrama amblycephala)	762,858	450	500	1,526	1,695	1,610
Japanese eel (Anguilla japonica)	265,759	146	249	1,067	1,823	1,445
Amur catfish (Silurus asotus)	360,050	250	250	1,440	1,440	1,440
Rainbow trout (Oncorhynchus mykiss)	916,540	507	896	1,022	1,809	1,416
Giant gourami (Osphronemus goramy)	206,059	200	300	687	1,030	858
Mrigal carp (Cirrhinus mrigala)	523,888	600	700	748	873	811
Gilthead seabream (Sparus aurata)	258,754	300	400	647	863	755
European seabass (Dicentrarchus labrax)	263,215	301	422	623	875	749
Largemouth black bass (Micropterus salmoides)	480,136	680	680	706	706	706
Blue-Nile tilapia, hybrid (Oreochromis aureus x O. niloticus)	410,553	603	603	681	681	681
Mandarin fish (Siniperca chuatsi)	337,082	500	500	674	674	674
Snakehead (Channa argus)	462,184	700	700	660	660	660
Large yellow croaker (Larimichthys croceus)	225,549	350	350	644	644	644
Climbing perch (Anabas testudineus)	59,496	90	100	595	661	628
Channel catfish (Ictalurus punctatus)	453,511	695	769	590	652	621
Africa-bighead catfish, hybrid (Clarias gariepinus x C. macrocephalus)	102,357	150	350	292	682	487
Atlantic salmon (Salmo salar)	2,615,962	4,719	6,364	411	554	483
North African catfish (Clarias gariepinus)	235,580	423	854	276	556	416
Japanese seabass (Lateolabrax japonicus)	184,020	368	600	307	500	403
Pompano (Trachinotus ovatus)	168,000	400	600	280	420	350
Snakeskin gourami (Trichogaster pectoralis)	30,098	80	120	251	376	314
Pirapatinga (Piaractus brachypomus)	206,981	500	1,000	207	414	310
Black carp (Mylopharyngodon piceus)	680,451	2,000	3,000	227	340	284
Mozambique tilapia (Oreochromis mossambicus)	74,435	300	303	245	248	247
Barramundi(=Giant seaperch) (Lates calcarifer)	107,868	322	2,096	51	335	193
Silver seabream (Pagrus auratus)	62,000	400	400	155	155	155
Striped snakehead (Channa striata)	69,531	300	1,000	70	232	151
Cachama (Colossoma macropomum)	104,140	500	1,985	52	208	130
Philippine catfish (Clarias batrachus)	16,288	125	125	130	130	130
Coho(=Silver) salmon (Oncorhynchus kisutch)	221,327	1,814	5,443	41	122	81
Japanese amberjack (Seriola quinqueradiata)	135,600	1,000	8,000	17	136	76
Bastard halibut (Paralichthys olivaceus)	45,320	400	1,500	30	113	72
Turbot (Scophthalmus maximus)	77,710	700	4,330	18	111	64
Indonesian snakehead (Channa micropeltes)	29,639	500	700	42	59	51
Red drum (Sciaenops ocellatus)	77,009	1,373	1,789	43	56	50
Korean rockfish (Sebastes schlegeli)	20,348	500	500	41	41	41
Crucian carp (Carassius carassius)	6,481	150	400	16	43	30
European eel (Anguilla anguilla)	5,496	150	600	9	37	23
Tambacu, hybrid (P. mesopotamicus x C. macropomum)	32,098	1,500	1,500	21	21	21
Tiger pufferfish (Takifugu rubripes)	21,588	832	1,500	14	26	20
Obscure pufferfish (Takifugu obscurus)	9,911	450	550	18	22	20
Meagre (Argyrosomus regius)	37,526	2,000	3,000	13	19	16
Mangrove red snapper (Lutjanus argentimaculatus)	7,387	500	500	15	15	15

Pangas catfish (Pangasius pangasius)	17,649	1,000	1,500	12	18	15
Pacu (Piaractus mesopotamicus)	15,606	1,200	1,200	13	13	13
European perch (Perca fluviatilis)	954	80	120	8	12	10
Snubnose pompano (Trachinotus blochii)	3,919	400	600	7	10	8
Cobia (Rachycentron canadum)	48,163	6,000	8,000	6	8	7
Flathead grey mullet (Mugil cephalus)	6,124	750	1,750	3	8	6
Striped bass, hybrid (Morone chrysops x M. saxatilis)	4,846	795	898	5	6	6
Brook trout (Salvelinus fontinalis)	1,702	219	560	3	8	5
Blue tilapia (Oreochromis aureus)	3,100	500	800	4	6	5
Senegalese sole (Solea senegalensis)	1,511	350	350	4	4	4
Chinook(=Spring=King) salmon (Oncorhynchus tshawytscha)	14,209	3,500	3,500	4	4	4
Japanese seabream (Pagrus major)	5,502	1,800	2,000	3	3	3
Pike-perch (Sander lucioperca)	3,171	1,000	2,000	2	3	2
Sea trout (Salmo trutta)	3,252	2,121	3,000	1	2	1
Pacific bluefin tuna (Thunnus orientalis)	26,057	19,000	28,000	1	1	1
Florida pompano (Trachinotus carolinus)	613	453	680	1	1	1
Marble goby (Oxyeleotris marmorata)	421	400	400	1	1	1
Orange-spotted grouper (Epinephelus coioides)	638	600	1,200	1	1	1
Siberian sturgeon (Acipenser baerii)	513	700	700	1	1	1
Atlantic halibut (Hippoglossus hippoglossus)	1,609	2,000	5,000	<1	1	1
Silver perch (Bidyanus bidyanus)	297	350	1,200	<1	1	1
Atlantic bluefin tuna (Thunnus thynnus)	12,130	30,000	50,000	<1	<1	<1
Atlantic cod (Gadus morhua)	900	2,000	5,000	<1	<1	<1
Striped bass (Morone saxatilis)	203	450	1,400	<1	<1	<1
Southern bluefin tuna (Thunnus maccoyii)	8,252	32,000	32,000	<1	<1	<1
Atipa (Hoplosternum littorale)	15	100	100	<1	<1	<1
Yellowtail amberjack (Seriola lalandi)	407	3,000	4,000	<1	<1	<1
American yellow perch (Perca flavescens)	24	238	321	<1	<1	<1
Brown-marbled grouper (Epinephelus fuscoguttatus)	43	600	1,200	<1	<1	<1
Common sole (Solea solea)	11	293	350	<1	<1	<1
Orbicular batfish (Platax orbicularis)	25	500	1,500	<1	<1	<1
Greater amberjack (Seriola dumerili)	109	3,000	5,000	<1	<1	<1
White-spotted spinefoot (Siganus canaliculatus)	0	100	150	<1	<1	<1
European whitefish (Coregonus lavaretus)	3	950	950	<1	<1	<1
Giant grouper (Epinephelus lanceolatus)	6	1,000	20,000	<1	<1	<1
**Total for single-species EMWs (91)**	**45,384,315**			**62,656**	**125,605**	**94,130**

Section 3 Single-species EMW numbers by species group

Note - this section calculates GEMWs to estimate numbers for species categories without an EMW, see Section 7 below.

Species group	Production (t)	Numbers lower (millions)	Numbers upper (millions)	Lower GEMW	Upper GEMW
Carps (Cyprinidae)	25,787,073	17,952	38,714	666	1,436
Pond loach	357,806	13,605	35,781	10	26
Tilapias (Cichlidae)	5,078,380	9,587	14,503	350	530
Catfishes (Siluriformes)	4,404,661	6,825	13,513	326	645
Perciformes	2,413,487	4,209	5,444	443	573
Milkfish (Chanidae)	1,536,970	3,620	6,148	250	425
Swamp & freshwater eels (Anguillidae & Synbranchidae)	585,126	2,645	4,998	117	221
Snakeheads & gouramies (Channidae, Osphronemidae, Anabantidae etc)	857,007	2,305	3,019	284	372
Salmon & trout (Salmonidae)	3,772,996	1,482	2,499	1,510	2,545
Others	590,809	426	986	599	1,388
	**45,384,315**	**62,656**	**125,605**		
		**Overall GEMW**		**361**	**724**

Section 4 Multi-species categories with an EMW

Note - EMWs for multi-species categories are calculated in Section 5

Species	Production (t)	Lower global EMW	Upper global EMW	Numbers lower (millions)	Numbers upper (millions)	Numbers midpoint (millions)
Carassius spp (Carassius spp)	2,755,687	150	500	5,511	18,371	11,941
Torpedo-shaped catfishes nei (Clarias spp)	1,260,107	125	854	1,475	10,081	5,778
Tilapias nei (Oreochromis (=Tilapia) spp)	1,099,860	300	530	2,074	3,666	2,870
Pangas catfishes nei (Pangasius spp)	531,000	933	1,500	354	569	461
Groupers nei (Epinephelus spp)	229,379	400	3,200	72	573	323
Silver, bighead carps nei (Hypophthalmichthys spp)	68,239	700	2,000	34	98	66
Sturgeons nei (Acipenseridae)	119,827	2,870	5,920	20	42	31
Snakeheads(=Murrels) nei (Channa spp)	10,350	300	1,000	10	35	22
Amberjacks nei (Seriola spp)	32,445	1,000	8,000	4	32	18
Trouts nei (Salmo spp)	18,559	2,121	6,364	3	9	6
Chars nei (Salvelinus spp)	1,829	219	560	3	8	6
Catfishes nei (Ictalurus spp)	1,299	695	769	2	2	2
River eels nei (Anguilla spp)	404	146	600	1	3	2
Snappers nei (Lutjanus spp)	674	500	500	1	1	1
Mugil spp (Mugil spp)	160	750	1,750	<1	<1	<1
Gouramis nei (Trichogaster spp)	10	80	120	<1	<1	<1
Pufferfishes nei (Takifugu spp)	21	450	1,500	<1	<1	<1
**Total for multi-species EMWs (17)**	**6,129,852**			**9,565**	**33,490**	**21,528**

Section 5 Multi-species EMWs

Note - multi-species EMWs are usually calculated from the range of the smallest & largest species farmed with size information available

Species	Species based on	Lower global EMW	Upper global EMW		
Carassius spp (Carassius spp)		150	500	-	-
Smallest species	Crucian carp	150	400		
Largest species	Prussian carp	250	500		
Tilapias nei (Oreochromis (=Tilapia) spp)		300	530	-	-
Smallest species	Mozambique tilapi	300	303		
Largest species	Nile tilapia	338	530		
Torpedo-shaped catfishes nei (Clarias spp)		125	854	-	-
Smallest species	Philippine catfish	125	125		
Largest species	North African catfish	423	854		
Pangas catfishes nei (Pangasius spp)		933	1,500	-	-
Smallest species	Striped catfish	933	1,385		
Largest species	Pangas catfish	1,000	1,500		
Groupers nei (Epinephelus spp)	Groupers generally	400	3,200		
Silver, bighead carps nei (Hypophthalmichthys spp)		700	2,000	-	-
Smallest species	Silver carp	700	1,500		
Largest species	Bighead carp	750	2,000		
Sturgeons nei (Acipenseridae)	Sturgeons generally	2,870	5,920		
Amberjacks nei (Seriola spp)		1,000	8,000	-	-
Smallest species	Japanese amberjack	1,000	8,000		
Largest species	Greater amberjack	3,000	5,000		
Snakeheads(=Murrels) nei (Channa spp)		300	1,000	-	-
Smallest species	Striped snakeheac	300	1,000		
Largest species	Striped snakeheac	300	1,000		
Trouts nei (Salmo spp)		2,121	6,364	-	-
Smallest species	Sea trout	2,121	3,000		
Largest species	Atlantic salmon	4,719	6,364		
Catfishes nei (Ictalurus spp)		695	769	-	-
Smallest species	Channel catfish	695	769		
Largest species	Channel catfish	695	769		
River eels nei (Anguilla spp)		146	600	-	-

Smallest species	Japanese eel	146	249		
Largest species	European eel	150	600		
Snappers nei (Lutjanus spp)		500	500	-	-
Smallest species	Mangrove red sna	500	500		
Largest species	Mangrove red sna	500	500		
Chars nei (Salvelinus spp)		219	560	-	-
Smallest species	Brook trout	219	560		
Largest species	Brook trout	219	560		
Mugil spp (Mugil spp)		750	1,750	-	-
Smallest species	Flathead grey mull	750	1,750		
Largest species	Flathead grey mull	750	1,750		
Gouramis nei (Trichogaster spp)		80	120	-	-
Smallest species	Snakeskin gouram	80	120		
Largest species	Snakeskin gouram	80	120		
Pufferfishes nei (Takifugu spp)		450	1,500	-	-
Smallest species	Obscure pufferfish	450	550		
Largest species	Tiger pufferfish	832	1,500		

Section 6 Species categories without an EMW

Species	Production (t)
Freshwater fishes nei (Osteichthyes)	2,515,482
Marine fishes nei (Osteichthyes)	751,907
Cyprinids nei (Cyprinidae)	679,488
Mullets nei (Mugilidae)	259,764
Porgies, seabreams nei (Sparidae)	101,823
Lefteye flounders nei (Bothidae)	49,809
Orangefin labeo (Labeo calbasu)	47,341
Nilem carp (Osteochilus hasselti)	43,800
Chinese longsnout catfish (Leiocassis longirostris)	22,063
Righteye flounders nei (Pleuronectidae)	16,006
Nile perch (Lates niloticus)	15,582
Stinging catfish (Heteropneustes fossilis)	15,528
Salmonids nei (Salmonidae)	14,192
Hoven's carp (Leptobarbus hoeveni)	13,901
Clearhead icefish (Protosalanx hyalocranius)	13,541
Fourfinger threadfin (Eleutheronema tetradactylum)	11,922
Tambatinga, hybrid (C. macropomum x P. brachypomus)	11,650
Sorubims nei (Pseudoplatystoma spp)	11,005
Pond smelt (Hypomesus olidus)	10,730
Arctic char (Salvelinus alpinus)	8,479
John's snapper (Lutjanus johnii)	7,243
Kissing gourami (Helostoma temminckii)	7,200
Asian redtail catfish (Hemibagrus nemurus)	7,119
Aba (Gymnarchus niloticus)	6,789
Asian barbs nei (Puntius spp)	6,517
Reticulate knifefish (Papyrocranus afer)	6,465
African bonytongue (Heterotis niloticus)	6,297
Ayu sweetfish (Plecoglossus altivelis)	6,025
Small scale mud carp (Cirrhinus microlepis)	6,000
Olive barb (Systomus sarana)	5,752
Manipur osteobrama (Osteobrama belangeri)	5,200
Brycon amazonicus (Brycon amazonicus)	4,911
Brycon spp (Brycon spp)	4,884
Three spotted tilapia (Oreochromis andersonii)	4,793
Whitefishes nei (Coregonus spp)	4,762
Characins nei (Characidae)	4,759
Tilapia shiranus (Oreochromis shiranus)	4,711
Upsidedown catfishes (Synodontis spp)	4,569
White trevally (Pseudocaranx dentex)	4,400
Grass-eaters nei (Distichodus spp)	4,348
Isok barb (Probarbus jullieni)	4,320
Tiger-dragon grouper, hybrid (E. fuscoguttatus x E. lanceolatus)	4,300
Streaked prochilod (Prochilodus lineatus)	4,226
Citharinus nei (Citharinus spp)	4,175
Dorada (Brycon moorei)	4,038
Mud carp (Cirrhinus molitorella)	4,013
Salmonoids nei (Salmonoidei)	3,784
Hetero-Clarias catfish, hybrid (H. longifilis x C. gariepinus)	3,610

Twobar seabream (Acanthopagrus bifasciatus)	3,560
Kafue pike (Hepsetus odoe)	3,550
Leporinus spp (Leporinus spp)	3,048
Redbreast tilapia (Tilapia rendalli)	2,999
Red porgy (Pagrus pagrus)	2,933
Parachanna snakeheads nei (Parachanna spp)	2,663
Magdalena River prochilodus (Prochilodus magdalenae)	2,452
Arapaima (Arapaima gigas)	2,154
Wels(=Som) catfish (Silurus glanis)	2,091
Longfin tilapia (Oreochromis macrochir)	1,800
Oreochromis tanganicae (Oreochromis tanganicae)	1,700
Brycon melanopterus (Brycon melanopterus)	1,548
Northern pike (Esox lucius)	1,542
Roaches nei (Rutilus spp)	1,399
Oplegnathus fasciatus (Oplegnathus fasciatus)	1,262
Freshwater bream (Abramis brama)	1,117
Spotted snakehead (Channa punctata)	1,100
Wallago (Wallago attu)	1,093
Blackhead seabream (Acanthopagrus schlegeli)	1,049
Bronze featherback (Notopterus notopterus)	898
Great snakehead (Channa marulius)	880
Trahira (Hoplias malabaricus)	808
Japanese jack mackerel (Trachurus japonicus)	800
Tench (Tinca tinca)	792
Filefishes, leatherjackets nei (Monacanthidae)	719
Banded astyanax (Astyanax fasciatus)	661
Spotted rose snapper (Lutjanus guttatus)	600
Jacks, crevalles nei (Caranx spp)	506
Black prochilodus (Prochilodus nigricans)	468
Longfin yellowtail (Seriola rivoliana)	400
Dotted gizzard shad (Konosirus punctatus)	390
Goldfish (Carassius auratus)	384
Murray cod (Maccullochella peelii)	360
Danube sturgeon(=Osetr) (Acipenser gueldenstaedtii)	311
Sabaki tilapia (Oreochromis spilurus)	300
Finfishes nei (Osteichthyes)	291
Spinefeet(=Rabbitfishes) nei (Siganus spp)	287
Pacific fat sleeper (Dormitator latifrons)	261
Pacific chub mackerel (Scomber japonicus)	230
Clown knifefish (Chitala chitala)	227
Peled (Coregonus peled)	221
Scorpionfishes, redfishes nei (Scorpaenidae)	207
Kelee shad (Hilsa kelee)	200
Masu(=Cherry) salmon (Oncorhynchus masou)	193
Barbel (Barbus barbus)	178
Snappers, jobfishes nei (Lutjanidae)	173
Malabar grouper (Epinephelus malabaricus)	150
Brycon cephalus (Brycon cephalus)	148
Convict grouper (Epinephelus septemfasciatus)	145
Mississippi paddlefish (Polyodon spathula)	144
Groupers, seabasses nei (Serranidae)	137
Blackspot(=red) seabream (Pagellus bogaraveo)	133
Shi drum (Umbrina cirrosa)	133
Long-finned charr (Salvethymus svetovidovi)	103
Roach (Rutilus rutilus)	100
Crimson snapper (Lutjanus erythropterus)	98
Labeo dussumieri (Labeo dussumieri)	96
Blackchin tilapia (Sarotherodon melanotheron)	95
Cichla spp (Cichla spp)	92
Black bullhead (Ameiurus melas)	90
Algerian barb (Barbus callensis)	87
Barcoo grunter (Scortum barcoo)	84
Bluespotted seabream (Pagrus caeruleostictus)	74
Dorado (Salminus brasiliensis)	72
Pink dentex (Dentex gibbosus)	66
Sterlet sturgeon (Acipenser ruthenus)	56
Brycon orbignyanus (Brycon orbignyanus)	55
Bagrid catfish (Chrysichthys nigrodigitatus)	50
Diadromous fishes nei (Osteichthyes)	49
Mi-iuy (brown) croaker (Miichthys miiuy)	47
Knifefishes (Notopterus spp)	45
Adriatic sturgeon (Acipenser naccarii)	44
Hypsibarbus spp (Hypsibarbus spp)	35
Streamer fish (Agrostichthys parkeri)	35

Sharpsnout seabream (Diplodus puntazzo)	34
Prochilodus mariae (Prochilodus mariae)	32
Spotted coralgrouper (Plectropomus maculatus)	30
Prochilods nei (Prochilodus spp)	30
Goldlined seabream (Rhabdosargus sarba)	29
Common dentex (Dentex dentex)	27
Soles nei (Soleidae)	20
Mango tilapia (Sarotherodon galilaeus)	19
Pimelodus spp (Pimelodus spp)	16
Flatfishes nei (Pleuronectiformes)	15
Beluga (Huso huso)	13
Rudd (Scardinius erythrophthalmus)	12
Tinfoil barb (Barbonymus schwanenfeldii)	12
Sobaity seabream (Sparidentex hasta)	11
Japanese meagre (Argyrosomus japonicus)	10
South American catfish (Rhamdia quelen)	10
White seabream (Diplodus sargus)	10
Leporinus obtusidens (Leporinus obtusidens)	10
Huchen (Hucho hucho)	9
Sampa (Heterobranchus longifilis)	8
Chum(=Keta=Dog) salmon (Oncorhynchus keta)	7
Emperor red snapper (Lutjanus sebae)	7
Redbelly tilapia (Tilapia zillii)	6
Golden-lined spinefoot (Siganus lineatus)	5
Bayad (Bagrus bajad)	5
Thai mahseer (Tor tambroides)	5
Bleak (Alburnus alburnus)	5
Jaguar guapote (Cichlasoma managuense)	4
Golden trevally (Gnathanodon speciosus)	3
Russell's snapper (Lutjanus russelli)	3
Burbot (Lota lota)	2
Mottled spinefoot (Siganus fuscescens)	2
Pink(=Humpback) salmon (Oncorhynchus gorbuscha)	2
Spotted seabass (Dicentrarchus punctatus)	1
Southern red snapper (Lutjanus purpureus)	1
Grayling (Thymallus thymallus)	1
Amazon sailfin catfish (Pterygoplichthys pardalis)	1
Golden perch (Macquaria ambigua)	1
Velvety cichlids (Astronotus spp)	1
Common nase (Chondrostoma nasus)	1
Giant trevally (Caranx ignobilis)	1
Barred sorubim (Pseudoplatystoma fasciatum)	0.4
Banded jewelfish (Hemichromis fasciatus)	0.3
Green humphead parrotfish (Bolbometopon muricatum)	0.2
	**4,812,913**

Section 7 Subtotals for species without an EMW by species group

Species group	Production (t)	Lower GEMW	Upper GEMW	Numbers lower (millions)	Numbers upper (millions)	Numbers midpoint (millions)
No species info	3,267,729	361	724	4,511	9,044	6,777
Carps (Cyprinidae)	820,554	666	1,436	571	1,232	902
Perciformes	158,704	443	573	277	358	317
Catfishes (Siluriformes)	67,258	326	645	104	206	155
Tilapias (Cichlidae)	16,520	350	530	31	47	39
Snakeheads & gouramies (Channidae & Helostomatidae)	11,843	284	372	32	42	37
Salmon & trout etc (Salmoniformes)	62,049	1,510	2,545	24	41	33
Freshwater eels (Anguillidae)	-	117	221	-	-	-
Others	408,255	599	1,388	294	681	488
**Species categories without an EMW (166)**	**4,812,913**			**5,845**	**11,651**	**8,748**

Source of production tonnage: FAO (2021).
Source of EMWs: present study.
