# Supplementary material for: Estimating global numbers of farmed fishes killed for food annually from 1990 to 2019
Source: Anim Welf. 2023 Feb 6;32:e12. doi: 10.1017/awf.2023.4 (PMC10936281; doi:10.1017/awf.2023.4)
Supplement: Supplementary file 1 [file S0962728623000040sup001.zip › S0962728623000040supp005.pdf]

Table S5 Estimated numbers of farmed fishes, & reported birds & mammals, killed for food 1990-2019

Year	Farmed fish production '000 tonnes[1]	Total estimated fish numbers (billions)			Tonnage as % of 1990 tonnage	Midpoint of numbers as % of 1990 midpoint	Numbers of birds & mammals (billions)[3]
		lower[2]	upper[2]	midpoint			
1990	8,676	9	18	13	100%	100%	31
1991	8,981	9	18	14	104%	104%	32
1992	9,908	10	20	15	114%	114%	34
1993	11,213	11	23	17	129%	127%	35
1994	13,051	13	26	19	150%	144%	37
1995	14,995	14	29	22	173%	164%	39
1996	16,872	16	33	25	194%	186%	40
1997	17,895	17	36	26	206%	198%	42
1998	18,507	18	37	27	213%	204%	43
1999	19,875	19	40	30	229%	223%	45
2000	20,813	20	42	31	240%	231%	47
2001	22,165	21	45	33	255%	247%	48
2002	23,516	23	48	35	271%	266%	50
2003	24,230	28	59	43	279%	324%	51
2004	26,307	30	63	47	303%	349%	52
2005	27,980	33	70	52	322%	386%	54
2006	29,801	36	76	56	343%	419%	55
2007	31,605	39	83	61	364%	456%	58
2008	34,281	42	89	66	395%	492%	61
2009	35,754	44	95	70	412%	521%	63
2010	37,745	49	103	76	435%	567%	64
2011	39,448	51	110	80	455%	602%	66
2012	42,336	56	122	89	488%	666%	67
2013	44,988	61	132	97	519%	724%	68
2014	47,215	64	140	102	544%	765%	70
2015	48,979	67	147	107	565%	802%	72
2016	51,043	72	158	115	588%	861%	74
2017	52,631	77	166	121	607%	910%	76
2018	54,431	76	167	121	627%	908%	79
2019	56,327	78	171	124	649%	932%	80

1 FAO (2021).
2 Estimated using the method described in text.
3 FAO (2020a).
