# Supplementary material for: Estimating global numbers of farmed fishes killed for food annually from 1990 to 2019
Source: Anim Welf. 2023 Feb 6;32:e12. doi: 10.1017/awf.2023.4 (PMC10936281; doi:10.1017/awf.2023.4)
Supplement: Supplementary file 1 [file S0962728623000040sup001.zip › S0962728623000040supp006.pdf]

Table S6 Scottish farmed Atlantic salmon 2002-2019

Year	Production ('000 t) [1]	Percent change since 2002	Numbers Slaught-ered[1] (millions)	Percent change since 2002	Mean Slaughter weight (kg)	Percent change since 2002
2002	145		35		4.1	
2003	170	17%	38	9%	4.4	8%
2004	158	9%	35	0.5%	4.5	9%
2005	130	-10%	29	-18%	4.5	9%
2006	132	-9%	28	-19%	4.6	13%
2007	130	-10%	29	-18%	4.5	10%
2008	129	-11%	29	-17%	4.4	8%
2009	144	-0.2%	31	-13%	4.7	15%
2010	154	7%	32	-9%	4.8	17%
2011	158	9%	33	-7%	4.9	18%
2012	162	12%	35	-1%	4.7	14%
2013	163	13%	33	-7%	5.0	22%
2014	179	24%	34	-2%	5.2	27%
2015	172	19%	35	0.05%	4.9	19%
2016	163	13%	36	2%	4.6	11%
2017	190	31%	37	4%	5.2	26%
2018	156	8%	29	-17%	5.4	30%
2019	204	41%	39	11%	5.2	28%

1 Source: MSS (2020). Production and numbers slaughtered in each calendar year.
