# Supplementary material for: Estimating global numbers of farmed fishes killed for food annually from 1990 to 2019
Source: Anim Welf. 2023 Feb 6;32:e12. doi: 10.1017/awf.2023.4 (PMC10936281; doi:10.1017/awf.2023.4)
Supplement: Supplementary file 1 [file S0962728623000040sup001.zip › S0962728623000040supp007.pdf]

Table S7 Changes in average slaughter weight of US farmed fishes between 2005, 2013 and 2018[1]

Species	Mean weight (g)			% Change in mean weight			Prod'n 2018 (t)	% Total t 2018[2]	Numbers 2018	Change due to mean weight[3]
	2005	2013	2018	2005-13	2013-18	2005-18				
Channel catfish	695	769	746	11%	-3%	7%	159,421	76%	213,692	-15,568
Rainbow trout	488	625	599	28%	-4%	23%	22,369	11%	37,353	-8,481
Striped bass, hybrid	795	856	898	8%	5%	13%	3,941	2%	4,386	-570
Red drum		1,353	1,573		16%	16%	3,245	2%	2,063	-334
Grass carp	1,601	1,292	955	-19%	-26%	-40%	462	0.2%	484	195
American yellow perch	238		321			35%	24	0.01%	75	-26
Total of above							**189,461**	**90%**	**258,053**	**-24,784**
Percent change										**-9.6%**

1 Source: USDA Census of Aquaculture 2005, 2013 and 2018 (USDA 2007, 2014, 2019). Mean weights are calculated from numbers and weight. Means weights and tonnages are converted from pounds. Only identifiable species with mean weight data for two or more years are shown.
2 US farmed fish production in 2018 totalled 210,150 tonnes FAO (2021).
3 Difference between numbers calculated as the 2018 tonnage divided by the 2018 mean weight and the same divided by the 2005 mean weight (or 2013 mean weight for red drum).
