# Supplementary material for: Estimating global numbers of farmed fishes killed for food annually from 1990 to 2019
Source: Anim Welf. 2023 Feb 6;32:e12. doi: 10.1017/awf.2023.4 (PMC10936281; doi:10.1017/awf.2023.4)
Supplement: Supplementary file 1 [file S0962728623000040sup001.zip › S0962728623000040supp008.pdf]

**Table S8 Farmed fish welfare law in 28 producing countries and the EU27***

| Rank in 2019 | Country | Percent of global farmed fish numbers (by estimate midpoint) % | Any fish welfare law? | Farmed fish welfare law | | | Law includes welfare codes for fish slaughter |
|---|---|---|---|---|---|---|---|
| | | | | Rearing | Transport | Slaughter | |
| 1 | China | 58 | No[1] | No[1] | No[1] | No[1] | No[1] |
| 2 | Indonesia | 12 | Yes[2,3] | Yes[2,3] | Yes[2,3] | Yes[2,3] | No[3,4] |
| 3 | India | 6 | Yes[5] | Yes[5] | Yes[5] | Yes[5] | No[6] |
| 4 | Bangladesh | 4 | No[7] | No[7] | No[7] | No[7] | No[7] |
| 5 | Vietnam | 3 | Yes[8] | Yes[8] | Yes[8] | Yes[8] | No[9] |
| 6 | Philippines | 2 | Yes[10] | Yes[10] | Yes[10] | Yes[10] | No[11] |
| 7 | Myanmar | 2 | Yes[12] | Yes[12] | No[12] | No[12] | No[12] |
| 8 | Egypt | 2 | ?[13] | ?[13] | No[14] | No[14] | No[14] |
| 9 | Thailand | 1 | Yes[15] | No[15] | No[15] | No[15] | No[15] |
| 10 | Turkey | 1 | Yes[16] | Yes[16] | Yes[16] | Yes[16] | No[17,18] |
| 11 | Cambodia | 1 | ?[19] | ?[19] | ?[19] | ?[19] | ?[19] |
| 12 | Brazil | 1 | ? | ?[20] | ?[20] | No[21] | No[22] |
| 13 | EU27 | 1 | Yes | Yes[23] | Yes[24] | Yes[25] | No[25,26] |
| 14 | Iran | 1 | No[27] | No[27] | No[27] | No[27] | No[27] |
| 15 | Nigeria | 0.5 | Yes[28] | Yes[28] | Yes[28] | Yes[28] | No[29] |
| 16 | Taiwan | 0.4 | Yes[30] | Yes[30] | Yes[30] | Yes[30] | No[30] |
| 17 | Japan | 0.3 | No[31] | No[31] | No[31] | No[31] | No[31] |
| 18 | Malaysia | 0.3 | Yes[32] | Yes[32] | Yes[32] | Yes[32] | No[33] |
| 19 | Colombia | 0.3 | Yes | Yes[34] | Yes[35] | Yes[36] | No[37] |
| 20 | South Korea | 0.2 | Yes[38] | No[38] | No[38] | No[38] | No[38] |
| 21 | USA (Federal) | | No[39] | No[39] | No[39] | No[39,40] | No[39] |
| | USA (States) | 0.2 | Some[41] | No[39] | No[39] | No[39] | No[39] |
| 22 | Norway | 0.2 | Yes | Yes[42] | Yes[42] | Yes[42,43] | Yes[43] |
| 23 | Peru | 0.2 | Yes[44] | Yes[44] | Yes[44] | Yes[44] | No[45] |
| 24 | Chile | 0.2 | Yes[46] | Yes[46] | Yes[46] | Yes[46] | No[47] |
| 37 | UK | 0.04 | Yes[48] | Yes[49] | Yes[50] | Yes[51] | No[51] |
| 39 | Canada (federal) | | ?[52] | No[52] | No[52] | No[52] | No[52] |
| | Canada provinces | 0.03 | Some[53] | Some[53] | Some[53] | Some[53] | |
| 48 | Australia (federal) | | No[54] | No[54] | No[54] | No[54] | No[54] |
| | Australia (states) | 0.02 | Some[55] | Some[55] | Some[55] | Some[55] | NSW?[55] |
| 73 | Switzerland | 0.005 | Yes[56] | Yes[56] | Yes[56] | Yes[56] | Yes[56] |
| 82 | New Zealand | 0.003 | Yes[57 58] | Yes[57] | Yes[57] | Yes[57,58] | Yes[58] |
| | Others | 2 | | | | | |
| | Total | 100 | | | | | |

* General notes.
This table, based on the authors' interpretation of law, indicates where some basic general welfare requirements in law exist that apply to fish species, specifically or as vertebrates or living creatures. For rearing, these may be based on the Five Freedoms or may be just a requirement to provide adequate feed. Most welfare laws for transport and slaughter comprise a requirement to avoid causing unnecessary suffering. All links were accessed on 12 August 2022.

---

[1] Source: Animal Protection Index 2020 – People's Republic of China. World Society for the Protection of Animals https://api.worldanimalprotection.org/country/china

[2] Husbandry and Animal Health Law Number 18 Year 2009, dated June 4, 2009. Indonesian Government. Applies to vertebrates and some invertebrates that can feel pain eg crabs. Article 66. Slaughtering and killing of animals shall be conducted properly so that the animals feel free from pain and fear http://extwprlegs1.fao.org/docs/pdf/ins98701.pdf

[3] Regulation of the Government of the Republic of Indonesia Number 95 of 2012. Chapter III animal welfare. As with Law 18, vertebrates and also invertebrates that can feel pain are covered. The principle of freedom (definition based on the Five Freedoms) shall be applied to…maintenance and care, transportation… slaughtering and killing…. This includes slaughter by ways that do not cause fear and stress, and ending suffering as soon as possible. There are no fish-specific requirements http://www.flevin.com/id/lgso/translations/JICA%20Mirror/english/4936_PP_95_2012_e.html

[4] Source: Animal Protection Index 2020 – Indonesia. World Society for the Protection of Animals https://api.worldanimalprotection.org/country/indonesia

[5] Cruelty to Animals Act 1960. Indian Government. Animal is defined as any living creature other than a human being. Treating animals cruelly includes subjecting them to unnecessary pain or suffering…conveying in a manner causing unnecessary pain or suffering…failing to provide sufficient food… Nothing in this section applies to killing an animal for food unless this was accompanied by the infliction of unnecessary pain or suffering https://legislative.gov.in/actsofparliamentfromtheyear/prevention-cruelty-animals-act-1960

[6] Source: Animal Protection Index 2020 – India. World Society for the Protection of Animals https://api.worldanimalprotection.org/country/india

[7] Animal Welfare Act, 2019 (Act No. 12 of 2019). In Bengali. This act excludes fishes. It does, however, permit any animals not specifically included in the act to be subsequently included by Government declaration http://bdlaws.minlaw.gov.bd/act-1292.html?hl=1

[8] Law on Animal Health 2015. Vietnam Government. This law specifically includes fishes, crustaceans and other aquatic animals. Article 21 states that organizations and individuals who rear aquatic animals shall (a) manage, care for, rear and transport animals in a manner suitable to each animal species and (b) minimize pain and fear for animals and treat them humanely in aquaculture, transportation, slaughter and disease prevention https://apps.fas.usda.gov/newgainapi/api/report/downloadreportbyfilename?filename=Vietnam%20National%20Assembly%20Passes%20Animal%20Health%20Law_Hanoi_Vietnam_10-7-2015.pdf

[9] Source: Animal Protection Index 2020 – Vietnam. World Society for the Protection of Animals https://api.worldanimalprotection.org/country/vietnam

[10] The revised rules for implementing the Animal Welfare Act (AWA) of 1998 Republic Act 8485 as amended by RA 10631. Philippines Government. 'Animal' is defined to include aquatic animals which are defined to include fishes and crustaceans. These rules shall be strictly construed in favour of protecting the Five Freedoms. Inhumane handling and improper stunning of animals for slaughter is defined as an act of cruelty. https://www.bai.gov.ph/index.php/laws-issuances/republic-act

[11] Administrative Order No 18 on the humane slaughter of animals for food, pursuant to the Animal Welfare Act of 1998, does not mention fish. Philippines Government https://www.bai.gov.ph/index.php/laws-issuances/administrative-order/category/87-ao-2008

[12] The Animal Health and Development Law 1993. Myanmar Government. Animal is defined as a 'domestic animal bred by man or captured for a certain purpose, and also includes the semen, ovum and embryo of the animal'. We assume this includes fishes. This law states that it is an offence, punishable by a fine, to wilfully ill-treat an animal; …to deprive one of food and water; or to neglect to treat an animal when sick or wounded http://extwprlegs1.fao.org/docs/pdf/mya36263.pdf

[13] Article 119 of the Agriculture Law Number 53 of 1966 states it is forbidden to exercise cruelty to animals (source: consolidated text of Agriculture Law No 53 of 1966 http://www.fao.org/faolex/results/details/en/c/LEX-FAOC153081 English version : http://extwprlegs1.fao.org/docs/pdf/egy9077.pdf). The cases to which this ban applies are given in Decree 27 (1967) and include torture or imprisonment of animals and improper feeding, watering and ventilation (source: Aidaros, H 2005. Global perspectives - the Middle East: Egypt. *Revue scientifique et technique (International Office of Epizootics), 24:* 589-596). It is not clear which species are covered

[14] Decree of the Minister of Agriculture No. 45 (1967) concerns slaughter. This does not appear to cover fish species (source: Aidaros, H 2005. Global perspectives - the Middle East: Egypt. *Revue scientifique et technique (International Office of Epizootics), 24:* 589-596) http://boutique.oie.int/extrait/aidaros589596.pdf

[15] The Cruelty Prevention and Welfare of Animals Act, B.E. 2557 (2014). Thailand Government. Under this law no person shall perform any act which is deemed an act of cruelty to animal without justification. Cruelty is an act, or failure to act, which causes an animal to suffer physically or mentally. The act covers animals used for food though it does not state the species covered and we assume that fishes are included. Killing an animal for food shall not be deemed cruelty http://extwprlegs1.fao.org/docs/pdf/tha181025.pdf

[16] Animal Protection Law no: 5199, 2004. Turkish Government. This law covers animals slaughtered for food purposes. It includes vertebrates and invertebrates in the definition of wild animals, which is the only place where species are defined in Article 3 which explains the terms. This law states that all animals have a right to life within the provisions of the law. It is a principle that animals will be cared for, fed, sheltered and transported under the conditions suited to their species. When slaughtered for food, this must be done in the least painful manner taking into account the special conditions required by religious rules. http://www.lawsturkey.com/law/5199-animal-protection-law

[17] After the 2004 Animal Protection Law in Turkey went into effect, two new regulations were issued (the Regulation on the Welfare and Protection of Animals during Transport and the Regulation on welfare of Farm Animals). At the end of the 2014 there were no subject specific legislations on slaughtering or any species-specific stipulations except for welfare standards for the protection of calves and laying hens. Source: ÖZEN A 2017 A Historical Overview of Turkey’s Animal Welfare Legislation. Kafkas Universitesi Veteriner Fakultesi Dergisi 23:6 (Abstract) https://doi.org/10.9775/kvfd.2016.17214

[18] Source: Animal Protection Index 2020 – Turkey. World Society for the Protection of Animals https://api.worldanimalprotection.org/country/turkey

[19] The Law on Animal Health and Production (No. NS/RKM/0116/003), dated 2016, has animal welfare as one of its objectives. Source FAOLEX database http://www.fao.org/faolex/results/details/en/c/LEX-FAOC173984/. An English translation was not obtained.

[20] Normative instruction 56 of 2008 applies to production animals in the various stages of the animal's life. Article 3 requires facilities to be designed to ensure animal welfare and unnecessary suffering to be avoided when transporting animals. The species covered are not defined. It also states that good practice recommendations for specific procedures for each animal species will be published https://www.gov.br/agricultura/pt-br/assuntos/sustentabilidade/bem-estar-animal/arquivos/arquivos-legislacao/in-56-de-2008.pdf (in Portuguese).

[21] Decree 30.691 of 1952 states (article 135) that butcher animals slaughtered for food must be killed by humane methods with prior stunning based on scientific principles http://extwprlegs1.fao.org/docs/pdf/bra157185.pdf (in Portuguese). Normative Instruction 3 of 2000 applies to butchery animals defined as some mammals and domestic birds and wild animals reared in captivity https://www.gov.br/agricultura/pt-br/assuntos/sustentabilidade/bem-estar-animal/arquivos/arquivos-legislacao/in-03-de-2000.pdf (in Portuguese). In Brazil, the Humane Slaughter Regulation 03/2000 excludes fish species. Even though the regulation is under review, ‘fish will not likely be included due to lack of scientific knowledge regarding proper stunning for the most commonly produced fish species’. Source: Souza APO, Leite LO and Molento CFM 2019 Animal welfare in Central and South America: What is going on? https://www.fondation-droit-animal.org/documents/9-SOUZA-et-al-AnimalWelfare2019.v1.pdf

[22] There are still no relevant regulations regarding welfare practices that take into account the welfare of fish. Source: Rodrigues DA, Junior AGC, Balista WC and de Freitas RR 2015. Animal welfare concerns at a fish farming operation in southeastern Brazil. Revista de Gestão Costeira Integrada-Journal of Integrated Coastal Zone Management 15: 417-424 https://www.redalyc.org/pdf/3883/388341160010.pdf

[23] EU Council Directive 98/58/EC of 20 July 1998 concerning the protection of animals kept for farming purposes. European Commission. This directive specifically includes fish and requires member states to make provision to ensure that the owners and keep take all reasonable steps to ensure the welfare of animals under their care and to ensure that those animals are not caused any unnecessary pain, suffering or injury https://eur-lex.europa.eu/legal-content/EN/TXT/?uri=celex%3A31998L0058

[24] EU Council Regulation (EC) No 1/2005 of 22 December 2004 on the protection of animals during transport and related operations and amending Directives 64/432/EEC and 93/119/EC and Regulation (EC) No 1255/97. European Commission. This regulation covers vertebrates and states that no person shall transport animals, or cause animals to be transported, in a way likely to cause undue suffering to them https://eur-lex.europa.eu/legal-content/en/TXT/?uri=CELEX%3A32005R0001

[25] EU COUNCIL REGULATION (EC) No 1099/2009 of 24 September 2009 on the protection of animals at the time of killing. European Commission. Article 3.1, which gives a general requirement to spare animals avoidable pain, distress or suffering; covers fish species but there are no detailed requirements for them https://eur-lex.europa.eu/legal-content/EN/ALL/?uri=CELEX:32009R1099

[26] Source: Report from the Commission to the European Parliament and the Council on the Possibility of Introducing Certain Requirements Regarding the Protection of Fish at the Time of Killing 2018. European Commission. In this report it was stated that the Commission considered it not appropriate to propose specific requirements on the protection of fish at the time of killing https://eur-lex.europa.eu/legal-content/EN/TXT/PDF/?uri=CELEX:52018DC0087&from=en.

[27] Source: Animal Protection Index 2020 – Islamic Republic of Iran. World Society for the Protection of Animals. The first animal protection law in Iran has been drafted but not yet enacted https://api.worldanimalprotection.org/country/iran

[28] Chapter 50 Cruelty to Animals. Laws of the Federation of Nigeria 1990. Under this law it is an offence to torture or to wantonly or unreasonably cause unnecessary suffering to any domestic or captive animal. It is an offence to cause unnecessary suffering transporting any animal. Nothing in this chapter applies to slaughtering an animal for food unless accompanied by the infliction of unnecessary suffering. Fish are specifically included in the definition of animal. There are no specific references to farm animal welfare http://lawsofnigeria.placng.org/laws/C38.pdf

[29] Source: Animal Protection Index 2020 – Nigeria. World Society for the Protection of Animals https://api.worldanimalprotection.org/country/Nigeria

[30] Animal Protection Law 1998 (amended 2021). Taiwan Government. This law covers vertebrates. Animals shall be prevented from panicking or suffering harm during transport. Anyone killing animals for food shall do so in a humane way to minimise animals' pain. Owners of animals must provide proper care including food https://law.moj.gov.tw/Eng/LawClass/LawAll.aspx?pcode=M0060027

[31] Act on Welfare and Management of Animals (Act No. 105 of October 1, 1973 https://www.env.go.jp/nature/dobutsu/aigo/1_law/files/aigo_kanri_1973_105_en.pdf ). Articles 2 and 7 require owners of animals to endeavour to maintain the health and safety of their animal. Article 40 states that where an animal must be destroyed, a method that minimizes as much as possible the pain and distress to the animal shall be used. However, fishes are not included in the definition of 'protected animal' in Article 44. Government guidance produced under this act includes guidance on rearing animals (Notification No. 22 of the Prime Minister's Office 1987 http://www.env.go.jp/hourei/18/000113.html in Japanese) and on slaughter (Notification No. 40 of the Prime Minister's Office 1995 http://www.env.go.jp/nature/dobutsu/aigo/2_data/laws/shobun.pdf in Japanese). While these appear mainly concerned with mammals and birds, they state that people should try to follow the same principles, which include that of pre-slaughter stunning to prevent pain, for all animals.

[32] Animal Welfare Act 2015. Under Section 24, owners have a duty to take reasonable steps to fulfil the needs of animals, including the need for a suitable diet and environment, and protection from pain and suffering. Nothing in this section applies to the lawful destruction of any animal for any reasonable purposes in an appropriate and humane manner. Section 27 provides some general animal welfare protections during transportation. The Act includes any living creature other than a human, including aquatic animals but excluding wildlife http://extwprlegs1.fao.org/docs/pdf/mal176896.pdf

[33] Under the National Animal Welfare Strategy 2012-2020, Codes of Practice detailing appropriate animal husbandry, transportation and slaughter were to be developed. These were not found in the analysis for the following source: Animal Protection Index 2020 – Malaysia. World Society for the Protection of Animals https://api.worldanimalprotection.org/country/malaysia

[34] Decree 2113 of 2017 defines 'production animal' as vertebrates or invertebrates destined for commercial production and has some requirements protecting their welfare during rearing https://www.suin-juriscol.gov.co/viewDocument.asp?id=30034356 (in Spanish)

[35] The transport or transfer of animals, obliges the person who performs it to use procedures that do not involve cruelty, ill-treatment, extreme fatigue or lack of rest, drink and food for them, says Article 27 of Law 84 of 1989 https://www.suin-juriscol.gov.co/viewDocument.asp?id=1628319 (in Spanish). We assume this law covers fish species since it has been amended by Law 1774 of 2016, which recognises animal sentience and gives some protection to vertebrates, with principles based on the Five Freedoms http://www.suin-juriscol.gov.co/viewDocument.asp?ruta=Leyes/30019637 (in Spanish).

[36] Decree 497 of 1973 regulates Law 5 of 1972 and defines mistreatment of animals. This includes not giving a quick death, free from prolonged suffering, to any animal whose extermination is necessary for consumption or not. It is the responsibility of the Animal Defenders Boards to avoid mistreatment of animals https://www.funcionpublica.gov.co/eva/gestornormativo/norma.php?i=8990 and https://www.funcionpublica.gov.co/eva/gestornormativo/norma.php?i=8991 (in Spanish).
We assume that Decree 497 covers fish species since vertebrates are covered by Law 1774 (see 35) and vertebrates and invertebrates are covered by Decree 2113 (see 34).

[37] Source: Animal Protection Index 2020 – Columbia. World Society for the Protection of Animals https://api.worldanimalprotection.org/country/Colombia

[38] Article 3 of the Protection of Animals Act 2017 gives principles that shall be observed which are based on the Five Freedoms. Article 10 states that no animal shall be slaughtered in a cruel or revolting manner, and shall be free from unnecessary pain, fear, or stress during slaughter. However, the act applies only to mammals, birds and animals prescribed by presidential decree https://www.fao.org/faolex/results/details/en/c/LEX-FAOC107509. Presidential Decree 28211 of 2017 extended the term 'animal' to include reptiles, amphibians, and fishes. Source: Alberro A 2019 The State of Modern South Korean Animal Cruelty Law: An Overview with Comparison to Relevant United States and Swiss Law and the Future. *Wash U Global Stud L Rev 18* p 665 https://openscholarship.wustl.edu/cgi/viewcontent.cgi?article=1689&context=law_globalstudies However, those kept for the purpose of human consumption are excluded https://www.law.go.kr/LSW/eng/engLsSc.do?y=0&x=0&menuId=1&query=Animal+Protection+Decree#liBgcolor3

[39] Currently, there are no federal or state laws protecting the welfare of farmed fish (source: Levenda K 2013 Legislation to protect the welfare of fish. *Animal L 20:* 119 https://www.animallaw.info/sites/default/files/lralvol20_1_119.pdf

[40] Fish, like poultry, are not covered by the federal Humane Slaughter Act 1978. Source: Animal Protection Index 2020 – USA. World Society for the Protection of Animals https://api.worldanimalprotection.org/country/USA

[41] Fishes are included in anti-cruelty law in some states eg, in New York, section 353-a of the Agriculture and Markets Law protects all companion animals from aggravated cruelty. See New York Consolidated Laws, Agriculture & Markets Law - AGM Article 26 | NY State Senate (nysenate.gov).

Florida law protects fishes from deliberate cruelty (828.02 and 828.12) but excludes aquatic animals from the requirements for humane slaughter (828.22 and 828.23): 2018 Florida Statutes Chapter 828 Animals: Cruelty; Sales; Animal Enterprise Protection https://www.flsenate.gov/Laws/Statutes/2018/Chapter828/All.
Illinois law protects all companion animals from aggravated cruelty https://www.ilga.gov/legislation/ilcs/ilcs3.asp?ActID=1717&ChapterID=41.
Alaskan law excludes fishes https://codes.findlaw.com/ak/title-11-criminal-law/ak-st-sect-11-81-900.html from its anti-cruelty provisions https://codes.findlaw.com/ak/title-11-criminal-law/ak-st-sect-11-61-140.html

[42]Animal Welfare Act 2009. Norwegian Government. This law specifically includes fishes as well as decapods, squid and octopi. Animals must be kept in an environment that gives good welfare; meets the specific needs of the species and allows them to perform stimulating activities. Transportation of animals must minimise stress. Farmed animals must be stunned prior to killing to ensure loss of consciousness, unless the killing method provides immediate unconsciousness https://www.regjeringen.no/en/dokumenter/animal-welfare-act/id571188/

[43] Decree No. 1250 of 2006 relative to slaughterhouses and processing facilities for aquaculture animals. Norwegian Government. Fish must be anesthetized before or at the same time as killing and be anesthetized until death occurs. Anaesthesia should be done by a suitable method that does not cause the fish significant stress or pain. This decree bans carbon dioxide stunning, though not live chilling with carbon dioxide (which is being phased out (EU Commission 2017)), and also bans use of salt and ammonia. Live cooling must be performed without too rapid temperature changes or too low a temperature.https://www.fao.org/faolex/results/details/en/c/LEX-FAOC067734/ (in Norwegian)

[44] Animal Protection and Welfare Law 30407. Applies to vertebrates. Article 14 states that, for the purposes of the application of this law, any species of vertebrate animals kept in captivity are recognized as sentient beings. Article 5 concerns duty of care. Article 16 implies a general requirement for welfare of farm animals during rearing, transport and slaughter. The sacrifice must cause instant death or immediate animal unconsciousness. The unhealthy raising and transport of farm animals is prohibited in Article 23 https://busquedas.elperuano.pe/normaslegales/ley-de-proteccion-y-bienestar-animal-ley-n-30407-1331474-1/ .

[45] Supplementary measures for the protection of farm animals to be established by the Ministries of Agriculture and Irrigation, Environment and Production (referred to in law 30407 Article 7) were not found in the analysis for the following source: Animal Protection Index 2020 – Peru. World Society for the Protection of Animals https://api.worldanimalprotection.org/country/peru

[46] Law 20380 on Animal Protection 2009. Chile Government. Article 3 states that any person who has an animal, in whatever capacity, must take care of it and provide adequate food and shelter. Article 4 states that animals must be transported under conditions that avoid mis-treatment or harm to their health. Article 11 states that, in the benefit and sacrifice of animals, rational methods should be used to avoid unnecessary suffering. We assume this law covers fish species since Article 13 indicates that the provisions of this law apply to aquatic animals and Article 19 refers to vertebrate animals https://www.bcn.cl/leychile/navegar?idNorma=1006858 (in Spanish).

[47] Article 13F of Paragraph 6 of Title II of Decree 430 of Law of 18.892 1989 states that aquaculture, ie industry, shall provide for rules for safeguarding animal welfare and procedures that avoid unnecessary suffering. Chile Government https://www.bcn.cl/leychile/navegar?idNorma=13315 (in Spanish). Decree 28 of 2013 gives stunning requirements for mammals and birds but does not cover fish species https://www.bcn.cl/leychile/navegar/imprimir?idNorma=1051388&idVersion=2013-05-30

[48] The Animal Welfare (Sentience) Act 2022 recognises the sentiency of vertebrates, cephalopods and decapods. UK Government https://www.legislation.gov.uk/ukpga/2022/22/enacted/data.pdf

[49] Animal Welfare Act 2006. UK Government. Applies to vertebrates. Owners are required to take reasonable steps to ensure the needs of animals are met, including needs for normal behaviour patterns and the need to be protected from pain and suffering https://www.legislation.gov.uk/ukpga/2006/45/data.pdf. The Welfare of Farmed Animals (England) Regulations 2007 give species-specific codes but these do not cover fishes https://www.legislation.gov.uk/uksi/2007/2078/made/data.pdf

[50] UK legislation originating from the EU (EU Council Regulation (EC) No 1/2005 of 22 December 2004 on the protection of animals during transport and related operations and amending Directives 64/432/EEC and 93/119/EC and Regulation (EC) No 1255/97. See 24

[51] UK legislation originating from the EU (EU Council Regulation (EC) No 1099/2009 of 24 September 2009 on the protection of animals at the time of killing). Article 3.1 covers fish species but there are no detailed requirements for them. See 25

[52] The Canadian Federal Criminal Code 1985 makes it an offence to wilfully permit to be caused "unnecessary pain, suffering or injury to an animal or a bird" (445.1) but does not define animal https://laws-lois.justice.gc.ca/pdf/c-46.pdf. The Health of Animals Act 1990 and the Safe Food for Canadians Act 2012 are the only federal laws covering the welfare of farm animals. Source: Animal Protection Index 2020 – Canada. World Society for the Protection of Animals https://api.worldanimalprotection.org/country/canada. Regulations for humane animal transport, under the Health of Animals Act 1990, appear not to cover fish species https://inspection.canada.ca/DAM/DAM-animals-animaux/STAGING/text-texte/humane_trans_reg_then_now_fact_sheet_1550526161243_eng.pdf. The Safe Food for Canadians Regulations SOR-2018/108 requires food animals to be rendered unconscious before slaughter (Part 6 Division 7 Subdivision F) but 'food animal' does not appear to include fishes https://laws-lois.justice.gc.ca/eng/regulations/SOR-2018-108/FullText.html. A code of recommended practice for farmed salmonids has been developed https://www.nfacc.ca/codes-of-practice/farmed-salmonids.

[53] Quebec Animal Welfare and Safety Act updated 2020 appears to cover farmed fishes and requires animals are kept in a suitable place with sufficient space and transported in a suitable manner. For slaughter, the method used must cause the animal a minimum of pain and anxiety and a rapid loss of consciousness http://legisquebec.gouv.qc.ca/en/pdf/cs/B-3.1.pdf.
The Yukon Animal Protection Act makes it an offence to cause distress to an animal, including fishes, though it exempts reasonable and generally accepted practices of animal slaughter provided that these practices are carried out in a humane manner https://legislation.yukon.ca/acts/anpr.pdf.
Fishes are excluded from the animal protection provisions of the Newfoundland Animal Health and Protection Act https://www.assembly.nl.ca/legislation/sr/statutes/a09-1.htm#14_

[54] State and territory governments are responsible for animal production and welfare laws and their enforcement https://www.agriculture.gov.au/animal/welfare/animal-welfare-in-australia. Aquatic Animal Welfare Guidelines have been published by the National Aquaculture Council of Australia.

[55] Queensland Animal Care and Protection Act specifically covers fishes, cephalopods and crustaceans. Killing an animal in an inhumane way or in a way that causes it not to die quickly is an animal cruelty offence. https://www.legislation.qld.gov.au/view/html/inforce/current/act-2001-064#sec.11
In New South Wales, under the Prevention of Cruelty to Animals Act 1979, which covers fishes and also covers crustaceans in restaurants, reasonable steps must be taken to alleviate pain inflicted on animals https://www.legislation.nsw.gov.au/view/html/inforce/current/act-1979-200#statusinformation. This state has a code of practice for humane harvesting of fish and crustaceans, though this is not adherent with OIE guidelines since it allows some fish species to be killed by immersion in ice slurry without prior stunning. https://www.dpi.nsw.gov.au/animals-and-livestock/animal-welfare/animal-care-and-welfare/other/companion-animal-files/humane-harvesting-of-fish-and-crustaceans. This reference does not state whether the part relating to finfish gives requirements or guidelines.
Northern Territory Animal Welfare Act 1999 covers fishes, and also crustaceans in restaurants, and makes it an offence to cause unnecessary suffering to animals or to take reasonable action to mitigate suffering. Reasonable action includes destroying an animal in a manner that causes a quick death without unnecessary suffering https://legislation.nt.gov.au/en/Legislation/ANIMAL-WELFARE-ACT-1999
In contrast, in South Australia, the Animal Welfare Act of 1985 specifically excludes fishes https://www.legislation.sa.gov.au/LZ/C/A/ANIMAL%20WELFARE%20ACT%201985/CURRENT/1985.106.AUTH.PDF.
Western Australia's Animal Welfare Act 2002 does not cover fishes. https://www.agric.wa.gov.au/animalwelfare/western-australias-animal-welfare-law.
The Tasmanian Animal Welfare Act covers vertebrates, and any other prescribed creature, and states that those in charge of an animal have a duty to take all reasonable measures to ensure the welfare of the animal https://www.legislation.tas.gov.au/view/whole/html/inforce/current/act-1993-063.

The Victoria Prevention of Cruelty to Animals Act 1986 covers fishes, and also lobsters, crabs and crayfish. Causing unreasonable pain or suffering is a cruelty offence. https://content.legislation.vic.gov.au/sites/default/files/08febf3f-607b-3f8f-b3df-92028dee2fc1_86-46aa095%20authorised.pdf
The ACT Animal Welfare Act 1992 covers fishes and cephalopods, and crustaceans intended for human consumption. Causing unnecessary pain or stress in a cruelty offence. https://www.legislation.act.gov.au/a/1992-45

[56] Animal Protection Ordinance, status as a 01.03.2018. Includes requirements for care and feeding. Prohibited practices include transport of live fish on ice or in iced water. This legislation states that vertebrates and decapods must be stunned before killing and that animals must be stunned in a manner that avoids pain and suffering and causes immediate loss of consciousness. However, cervical dislocation is included as a permitted stunning method for fishes which is not adherent with OIE guidelines. The other permitted methods for fishes are percussive stunning, electrical stunning and mechanical destruction of the brain, which are all recommended by the OIE https://www.blv.admin.ch/blv/en/home/tiere/tierschutz.html.

[57] Animal Welfare Act 1999 https://www.legislation.govt.nz/act/public/1999/0142/latest/DLM49664.html.

[58] Commercial slaughter code of welfare 01.10.2018. This requires that eels must be rendered insensible for the duration of desliming, or killed beforehand, and that gill arches must not be ripped or severed in un-stunned finfish. Killing methods must result in a rapid and irreversible loss of consciousness. However, chilling followed by emersion (asphyxiation in air) appears to be permitted without proper stunning https://www.mpi.govt.nz/dmsdocument/46018-Code-of-Welfare-Commercial-slaughter. Has some requirements for crustaceans.
