# Supplementary material for: Estimating global numbers of farmed fishes killed for food annually from 1990 to 2019
Source: Anim Welf. 2023 Feb 6;32:e12. doi: 10.1017/awf.2023.4 (PMC10936281; doi:10.1017/awf.2023.4)
Supplement: Supplementary file 1 [file S0962728623000040sup001.zip › S0962728623000040supp009.pdf]

Estimated finfish numbers (millions) in annual certified aquaculture production (2013-2015)

Scheme[1]	Species in scheme[2]	Species estimate based on[3] (if different)	Certified production '000 t[4]	Number (lower) in millions[5]	Number (upper) in millions[5]	Numbers (midpoint) in millions
GLOBALGAP	Atlantic Salmon		1,655	260	351	305
GLOBALGAP	European Seabass		75	177	248	213
GLOBALGAP	Gilthead Seabream		58	144	193	169
GLOBALGAP	Rainbow Trout		91	101	180	140
GLOBALGAP	Pangasius	Striped catfish	103	74	110	92
GLOBALGAP	Other finfishes		5	2	2	2
GLOBALGAP	**Total for scheme**		**1,986**	**759**	**1,084**	**921**
ASC	Tilapia	Nile tilapia	148	279	437	358
ASC	Pangasius	Striped catfish	145	104	155	130
ASC	Salmon	Atlantic salmon	341	54	72	63
ASC	Trout	Rainbow trout	7	8	13	10
ASC	**Total for scheme**		**640**	**444**	**678**	**561**
GAABAP	Tilapia	Nile tilapia	140	263	413	338
GAABAP	Salmon	Atlantic salmon	397	62	84	73
GAABAP	Pangasius	Striped catfish	39	28	42	35
GAABAP	Other finfishes		2	3	3	3
GAABAP	**Total for scheme**		**577**	**357**	**541**	**449**
FOS	Rainbow Trout		87	97	172	134
FOS	European Seabass		32	76	107	92
FOS	Gilthead Seabream		25	62	83	72
FOS	Sea Bream	Gilthead seabream	5	12	17	14
FOS	Pangasius	Striped catfish	7	5	8	7
FOS	Atlantic Salmon		10	2	2	2
FOS	Other finfishes		21	11	20	16
FOS	Excluded from estimate		164	-	-	-
FOS	**Total for scheme**		**352**	**266**	**409**	**337**
Organic	Seabass	European seabass	1	3	4	3
Organic	Salmon	Atlantic salmon	16	3	3	3
Organic	Rainbow Trout		1	2	3	2
Organic	Other finfishes		4	5	9	7
Organic	**Total for scheme**		**23**	**12**	**19**	**15**
	**Total all schemes[6]**		**3,579**	**1,838**	**2,731**	**2,284**

Totals by species[6]

Species	Certified production '000 t	Number (lower) in millions	Number (upper) in millions	Numbers midpoint	Total 2015 production for species estimate based on[7] '000 t	Certified % of total 2015 tonnage
Tilapia	287	542	850	696	4,051	7%
Atlantic salmon/salmon	2,419	380	513	446	2,381	102%
European seabass/seabass	108	256	360	308	163	66%
Rainbow trout/trout	186	208	367	287	751	25%
Pangasius	293	212	314	263	2,083	14%
Gilthead seabream/seabream	88	219	292	255	169	52%
Other finfishes	33	20	35	28	-	
Excluded from estimate	164	-	-		-	
	**3,579**	**1,838**	**2,731**	**2,284**	**9,598**	
2015 total estimate (billions)		67	147	107		
		3%	2%	2%		

Notes

1 FOS = Friend of the Sea; GAABAP = Global Aquaculture Alliance Best Aquaculture Practices; ASC = Aquaculture Stewardship Council.

2 'Other finfishes' include meagre, turbot, carp, red drum, striped bass, catfish, Senegalese sole and Siberian sturgeon. FOS figures for arctic char, brook trout, Atlantic cod, yellowtail amberjack and several species of sturgeon were not included because they exceed total production for 2015 as reported by the FAO. These are shown as 'Excluded from estimate'.

3 This column indicates the species on which the estimate is based, where the precise species is not given in Potts et al (2016). For example, estimates for 'salmon' are based on a global EMW for Atlantic salmon.

4 Source of certified tonnages: Potts et al (2016). Includes data for 2013 (GAABAP, Organic), 2014 (FOS) & 2015 (GLOBALGAP, ASC). Note that certified production for organic schemes in 2013 and GLOBALGAP in 2015, additionally included respectively 160 and 21 thousand tonnes for unnamed species. These were not included in the estimate but may include fish species.

5 Estimated numbers are based on global EMWs for 2019.

6 It is assumed that farms are certified in one scheme only.

7 Source: FAO (2021). Total production in 2015 of the species on which the estimate is based eg for 'Tilapia' this is the total global production of Nile tilapia, including certified and uncertified. Total production shown for 'Atlantic salmon/salmon', ie total production for Atlantic salmon, is less than the certified salmon production, which therefore appears too large. This might be because certified salmon production includes other species of salmon eg coho salmon (*Oncorhynchus kisutch*). It might also indicate that the figure calculated for total certified salmon is overstated due to double certification.

This table shows estimated numbers of finfishes reared within certification schemes. These are calculated from certified production tonnages, obtained from Potts et al (2016), and estimated mean fish weights from the present study. Only finfish tonnages are included. The estimate assumes no double certification of fish farms. Some assumptions are also made for the precise species included in certified tonnages. Since the precise species composition is not known, an accurate calculation of the percentage of certified to total, ie certified and uncertified, production (last column in lower table) is not possible.
