# Supplementary material for: Estimating global numbers of farmed fishes killed for food annually from 1990 to 2019
Source: Anim Welf. 2023 Feb 6;32:e12. doi: 10.1017/awf.2023.4 (PMC10936281; doi:10.1017/awf.2023.4)
Supplement: Supplementary file 1 [file S0962728623000040sup001.zip › S0962728623000040supp010.pdf]

Table S10 Farmed fish mortalities

	Species	Aquaculture	Experimental	FAO stocking model[1]	Mass mortalities
1	Pond loach (Misgurnus anguillicaudatus)	High larval and juvenile mortality (Gao et al 2014)			Yanohara and Kagei 1983 cited in Milton et al 2018
2	Nile tilapia (Oreochromis niloticus)	Grow out phase 25-60% (Rana and Hasan 2013), Traditional polyculture 6% (FAO 1980)	22-71% (Abdalla et al 1996), 1-19% (Abdelghany and Ahmad 2002), 32-72% (Trọng et al 2013), 55-61% (Muyot et al 2018)		Abu-Elala et al 2016
3	Crucian carp (Carassius carassius)	Nursery ponds 10-30% and fingerling ponds 10-20% (FAO 2004-2021a), 5% (Yuan Xihe 2016)		20-30% (Yingwu 1989)	Fichi et al 2016
4	Common carp (Cyprinus carpio)	Nursery stage 30-60% and fingerling stage 50-75% (FAO 2004-2021b), Traditional polyculture 20% (FAO 1980)	19-49% (Nielsen et al 2010), 3-18% (Abdelghany and Ahmad 2002), 11-13% (Buck and Rose 1977)	20% (Yingwu 1989)	Ye et al 2017
5	Silver carp (Hypophthalmichthys molitrix)	Traditional polyculture 6% (FAO 1980), 5% (Yuan Xihe 2016)	14-17% (Khan et al 2009), 15-30% (Abdelghany and Ahmad 2002), 8-18% (Buck and Rose 1977)	2-15% (Yingwu 1989)	Shagar and El-Refaee 2012
6	Milkfish (Chanos chanos)	Fry to fingerling 30% (FAO 2007-2021), Rearing 54% (Smith 1981) and 30% (Chen 1952 cited in Bagarinao 1991)	18-22% (Muyot et al 2018)		Cruz-Lacierda et al 2004
7	Yellow catfish (Pelteobagrus fulvidraco)				Geng et al 2014
8	Silver barb (Barbonymus gonionotus)		10-18% (Sarker et al 2016), 7-13% (Mollah et al 2011)		
9	Striped catfish (Pangasius hypophthalmus)	Up to 30% early to mid months of production cycle and < 10% in later months (Phan et al 2009), 38-40% (Rana and Hasan 2013)	3-5% (Khan et al 2009), 10-20% (Mehboob et al 2017)		Yuasa et al 2003
10	Asian swamp eel (Monopterus albus)		Grow out 8-27% (Khanh and Ngan 2010)		Liu et al 2019
11	Bighead carp (Hypophthalmichthys nobilis)	Nursery stage 20-30% and fingerling stage <5% (FAO 2004-2021c), Traditional polyculture 3% (FAO 1980)		2-15% (Yingwu 1989)	
12	Wuchang bream (Megalobrama amblycephala)	Traditional polyculture 29% (FAO 1980), 10% (Yuan Xihe 2016)		10-20% (Yingwu 1989)	

[1] The expected mortality for a stocking model which is a suggested combination of species at specific stocking densities that will, with effective rearing management, be productive (Yingwu 1989)

References

Abdalla AA, McNabb CD and Batterson TR 1996 Ammonia dynamics in fertilized fish ponds stocked with Nile Tilapia. *The Progressive Fish-Culturist* 58:2 pp 117-123 (Abstract).

Abdelghany AE and Ahmad MH 2002 Effects of feeding rates on growth and production of Nile tilapia, common carp and silver carp polycultured in fertilized ponds. *Aquaculture Research* 33:6 pp 415-423.

Abu-Elala NM, Abd-Elsalam RM, Marouf S, Abdelaziz M and Moustafa M 2016 Eutrophication, ammonia intoxication, and infectious diseases: interdisciplinary factors of mass mortalities in cultured Nile tilapia. *Journal of aquatic animal health* 28:3 pp187-198 (Abstract).

Bagarinao TU 1991. *Biology of milkfish (Chanos chanos Forsskal)* Aquaculture Department, Southeast Asian Fisheries Development Center.

Buck DH, Baur RJ and Rose CR, 1977. Experiments in the recycling of swine manure using a polyculture of Asian and North American fishes. In *Agriculture and Energy* pp 385-394. Academic Press.

Cruz-Lacierda ER, Maeno Y, Pineda AJT and Matey VE 2004 Mass mortality of hatchery-reared milkfish (Chanos chanos) and mangrove red snapper (Lutjanus argentimaculatus) caused by Amyloodinium ocellatum (Dinoflagellida). *Aquaculture* 236:1-4 pp 85-94.

FAO 1980 Freshwater aquaculture development in China. Report of the FAO/UNDP study tour organized for French-speaking African countries. 22 April – 20 May 1980. FAO Fisheries Technical Paper 215 Accessed Freshwater aquaculture development in China (fao.org) 1st March 2021.

FAO 2004-2021a Cultured Aquatic Species Information Programme. Carassius carassius. Text by Weimin, M. In: FAO Fisheries Division. Rome. Updated 1 January 2004. Accessed http://www.fao.org/fishery/culturedspecies/Carassius_carassius/en 1st March 2021.

FAO 2004-2021b Cultured Aquatic Species Information Programme. Cyprinus carpio. Text by Peteri, A. In: FAO Fisheries Division. Rome. Updated 1 January 2004. Accessed http://www.fao.org/fishery/culturedspecies/Carassius_carassius/en 1st March 2021.

FAO 2004-2021c Cultured Aquatic Species Information Programme. Hypophthalmichthys nobilis. Text by Weimin M. In: FAO Fisheries Division [online]. Rome. Updated 1 January 2004. Accessed http://www.fao.org/fishery/culturedspecies/Hypophthalmichthys_nobilis/en 1st March 2021.

FAO 2007-2021 Cultured Aquatic Species Information Programme. Chanos chanos.Text by Nelson AL and Marygrace CQ. In: FAO Fisheries Division. Rome. Updated 23 November 2007. Accessed http://www.fao.org/fishery/culturedspecies/Chanos_chanos/en 1st March 2021.

Fichi G, Susini F, Cocumelli C, Cersini A, Salvadori M, Guarducci M and Cardeti G 2016 New detection of Cyprinid herpesvirus 2 in mass mortality event of Carassius carassius (L.), in Italy. *Journal of fish diseases* 39:12 pp1523-1527.

Gao L, Duan M, Cheng F and Xie S 2014 Ontogenetic development in the morphology and behavior of loach (Misgurnus anguillicaudatus) during early life stages. *Chinese Journal of Oceanology and Limnology* 32:5 pp 973-981 (Abstract).

Geng Y, Liu D, Han S, Zhou Y, Wang KY, Huang XL, Chen DF, Peng X and Lai WM 2014 Outbreaks of vibriosis associated with Vibrio mimicus in freshwater catfish in China. *Aquaculture*, *433*, pp.82-84. (Abstract).

Khan S, Hossain MS and Haque MM 2009 Effects of feeding schedule on growth, production and economics of pangasiid catfish (Pangasius hypophthalmus) and silver carp (Hypophthalmichthys molitrix) polyculture. *Journal of the Bangladesh Agricultural University* 7:1 pp 175-181 (Abstract).

Khanh, NH and Ngan HTB 2010 Current practices of rice field eel Monopterus albus (zuiew, 1973) culture in Vietnam. *Aquaculture Asia Magazine* 15:3.

Liu W, Fan Y, Li Z, Zhao J, Zhou Y, Jiang N, Zeng J, Cain K and Zeng L 2019 Isolation, identification, and classification of a novel rhabdovirus from diseased Chinese rice-field eels (Monopterus albus). *Archives of virology* 164:1 pp 105-116.

Mehboob A, Khan N, Atiq U, Iqbal KJ, Tayyab R, Batool SS, Batool HS, Amjad S and Tanveer M 2017 Effect of Fenugreek as a Feed Additive on the Growth, Body Composition and Apparent Nutrients Digestibility of Striped Catfish Pangasius hypophthalmus Fry. *Pakistan Journal of Zoology* 49:6.

Milton J, Paray BA and Rather IA 2018 A review on the biology and physiology of loach Misgurnus anguillicaudatus in China.

Mollah MFA, Moniruzzaman M and Rahman MM 2011 Effects of stocking densities on growth and survival of Thai Sharpunti (Barbonymus gonionotus) in earthen ponds. *Journal of the Bangladesh Agricultural University* 9:2 pp 327-338.

Muyot FB, Mutia MTM and Caunan PJH 2018 Growth performance and cost efficiency of tilapia (Oreochromis niloticus) and milkfish (Chanos chanos) fed extruded floating and non-floating feeds reared in net cages in Taal Lake. *Philippine Journal of Fisheries* 25:2 pp 41-56.

Nielsen HM, Ødegård J, Olesen I, Gjerde B, Ardo L, Jeney G and Jeney Z, 2010 Genetic analysis of common carp (Cyprinus carpio) strains: I: Genetic parameters and heterosis for growth traits and survival. *Aquaculture* 304:1-4 pp 14-21 (Abstract).

Phan LT, Bui TM, Nguyen TT, Gooley GJ, Ingram BA, Nguyen HV, Nguyen PT and De Silva SS 2009 Current status of farming practices of striped catfish, Pangasianodon hypophthalmus in the Mekong Delta, Vietnam. *Aquaculture 296:3-4* pp 227-236.

Rana K and Hasan M 2013 On-farm feeding and feed management practices for sustainable aquaculture production: an analysis of case studies from selected Asian and African countries. In: Hasan M and New M (eds). *On-farm feeding and feed management in aquaculture* pp 21-67. FAO Fisheries and Aquaculture Technical Paper No 583.

Sarker B, Rahman M and Amin MR 2016 Effects of stocking density on growth and production of silver barb (Barbonymus gonionotus) in pond. *The Agriculturists* 14:2 pp 61-66 (abstract).

Shagar GE and El-Refaee AM 2012 Studies on cultured silver carp (Hypophthalmichthys Molitrix) diseases induced by some bacterial, fungal and parasitic pathogens in Sharkia Governorate. *Journal of The Arabian Aquaculture Society Studies* 7:2 (Abstract).

Smith IR 1981 The economics of the milkfish fry and fingerling industry of the Philippines.

Trọng TQ, Mulder HA, van Arendonk JA and Komen H 2013 Heritability and genotype by environment interaction estimates for harvest weight, growth rate, and shape of Nile tilapia (*Oreochromis niloticus*) grown in river cage and VAC in Vietnam. *Aquaculture* 384 pp119-127.

Ye L, Lu M, Quan K, Li W, Zou H, Wu S, Wang J and Wang G 2017 Intestinal disease of scattered mirror carp Cyprinus carpio caused by Thelohanellus kitauei and notes on the morphology and phylogeny of the myxosporean from Sichuan Province, southwest China. *Chinese journal of oceanology and limnology* 35:3 pp 587-596 (Abstract).

Yingwu F 1989 Chapter 5 Pond culture of food fish. In: NACA *Integrated Fish Farming in China*. NACA Technical Manual 7. A World Food Day Publication of the Network of Aquaculture Centres in Asia and the Pacific, Bangkok, Thailand. Accessed Integrated fish farming in china (Fao.Org) 1st March 2021.

Yuan Xihe 2016 Is fish farming profitable in 2017? 2017 fish farming prospects and market price analysis. In Chinese. Accessed https://m.zhifure.com/snzfj/33794.html 1st March 2021.

Yuasa K, Kholidin EB, Panigoro N and Hatai K 2003 First isolation of Edwardsiella ictaluri from cultured striped catfish Pangasius hypophthalmus in Indonesia. *Fish Pathology* 38:4 pp 181-183 (Abstract).
